# Nest Etiquette—Where Ants Go When Nature Calls

**DOI:** 10.1371/journal.pone.0118376

**Published:** 2015-02-18

**Authors:** Tomer J. Czaczkes, Jürgen Heinze, Joachim Ruther

**Affiliations:** Biologie I, Universität Regensburg, Regensburg, Bavaria, Germany; University of Paris 13, FRANCE

## Abstract

Sanitary behaviour is an important, but seldom studied, aspect of social living. Social insects have developed several strategies for dealing with waste and faecal matter, including dumping waste outside the nest and forming specialised waste-storage chambers. In some cases waste material and faeces are put to use, either as a construction material or as a long-lasting signal, suggesting that faeces and waste may not always be dangerous. Here we examine a previously undescribed behaviour in ants – the formation of well-defined faecal patches. *Lasius niger* ants were housed in plaster nests and provided with coloured sucrose solution. After two months, 1–4 well defined dark patches, the colour of the sucrose solution, formed within each of the plaster nests. These patches never contained other waste material such as uneaten food items, or nestmate corpses. Such waste was collected in waste piles outside the nest. The coloured patches were thus distinct from previously described ‘kitchen middens’ in ants, and are best described as ‘toilets’. Why faeces is not removed with other waste materials is unclear. The presence of the toilets inside the nest suggests that they may not be an important source of pathogens, and may have a beneficial role.

## Introduction

Disease is an important challenge for animals, and especially so for social animals. Social insects such as ants and bees live in very confined spaces with many conspecifics, and are thus especially vulnerable to contagion. An important source of contagious material may be refuse and faecal matter, as they may serve as a substrate for or source of pathogenic micro-organisms [1,2], and many insects have developed elaborate ways of handling their faeces [3]. Social insects have developed systems of safely dealing with such hazardous material. For example, honey bees perform defecation flights to void faecal matter. Such defecation flights are even made by very young bees, which otherwise do not leave the hive [4]. Some social spider mites create a localised faecal pile just inside the entrance to their silk shelter [5,6]. Many ants form a refuse pile outside the nest, or deposit waste and faecal material in a special chamber, often termed a 'kitchen midden' [7–9]. Division of labour has even been shown in relation to sanitation in ants, with specialised refuse workers being the only ants to enter the refuse chamber [10–12], although such division of labour has only been observed in leaf-cutting ants. Ants perform a variety of other sanitary behaviours unrelated to faeces, such as sterilising brood exposed to pathogenic fungal spores with formic acid [13], or production and application of antimicrobial glandular secretions [14–16]. All such sanitary behaviours are no doubt costly, as they require either the transport of infectious material, the digging of special refuse chambers, the ‘sacrifice’ of specialised refuse workers, or the synthesis of glandular secretions [16].

However, faeces and waste products are not necessarily dangerous. Many insects live in close proximity to their faeces and suffer no ill effects [3]. Indeed, faeces may be put to use, for example as a defensive shield in beetle larvae, fertilizer in leaf cutter ants, or as a building or antimicrobial material in termites [17–20].

While examining laboratory nests, the authors noted that the ant *Lasius niger* seemed to form distinct dark patches within their nests. We suspected these to be faecal patches. These patches were very spatially defined, and were distinct from the refuse piles outside the nest as they almost never contained food remains, nest-mate corpses, or other refuse. In this study we examined this behaviour in a controlled manner to ascertain whether these dark patches were indeed faecal patches, and where in the nest these toilets were formed.

## Methods

**Fig 1:**
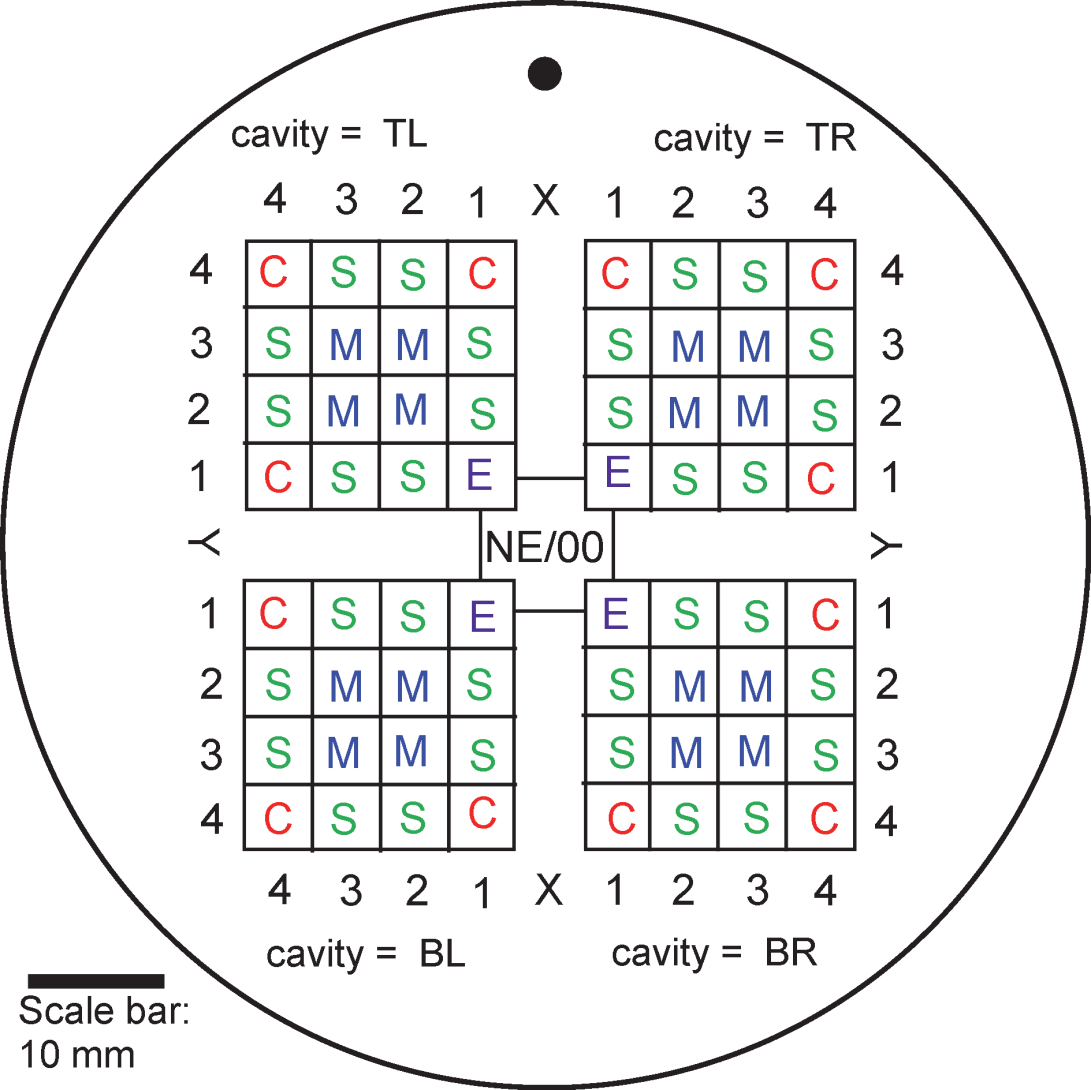
A schematic of the plaster nests in which the ants were housed. Access to the nest was via a 5mm diameter hole in the lid of the nest, at the centre of the nest entrance (NE/00) cavity. The locations of faecal patches (= toilets) were defined by their square type (C = corner, S = side, M = middle, E = cavity entrance, NE = nest entrance), and by an XY coordinate, with the nest entrance as an origin. Cavities are denoted TL, TR, BL, BR (Top left, top right, etc.) according to their location relative to the side of the nest facing away from the nearest wall. The plaster nest was placed to one side of a square foraging box. The black dot represents the side of the nest facing away from the nearest wall of the foraging box

For a detailed methodology, see S1 Methods. In brief, 21 small colonies of ants were housed in plaster nests (Fig. 1), placed in a foraging box. The ants were fed on coloured sucrose solution and a differently coloured protein source. Pictures of the nests and foraging boxes were taken once a week for two months. After two months the ants were removed, and using an uninformed observer the location of any “darker or coloured patches” were recorded (see S1 Methods for the instructions provided to the blind observer).

## Results

**Fig 2:**
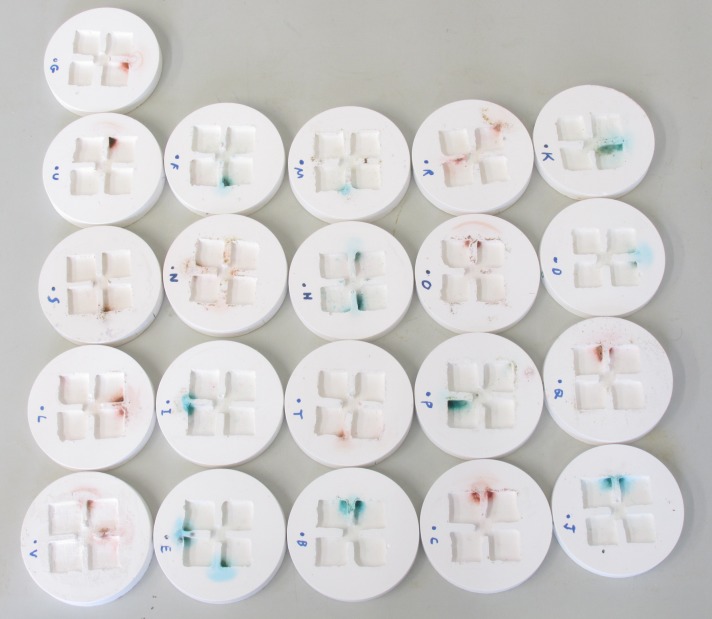
Ant toilets. 21 plaster nests which had been inhabited by 150–300 *Lasius niger* workers for 2 months. Dark coloured patches (= toilets) can be seen in every nest. The colour of the patch corresponds to the colour of the sugar solution the ants were fed. High resolution images of each nest and the surrounding foraging arena, and images taken throughout the course of the experiment, are available from the Dryad Digital Repository: http://dx.doi.org/10.5061/dryad.[doi:10.5061/dryad.9fs7n].

Distinct, localised, coloured patches formed in all 21 nests (Fig. 2). The patches were always the same colour as the sugar solution presented. Between 1 and 4 patches were formed in each nest (mean 2.32, median 2). In total 41 such patches were formed. No trace of the protein colour was found in the patches. Adult worker ants require little or no protein, and consumption of proteins or amino-acids by adult ants can be lethal to adult workers [21,22].

**Fig 3:**
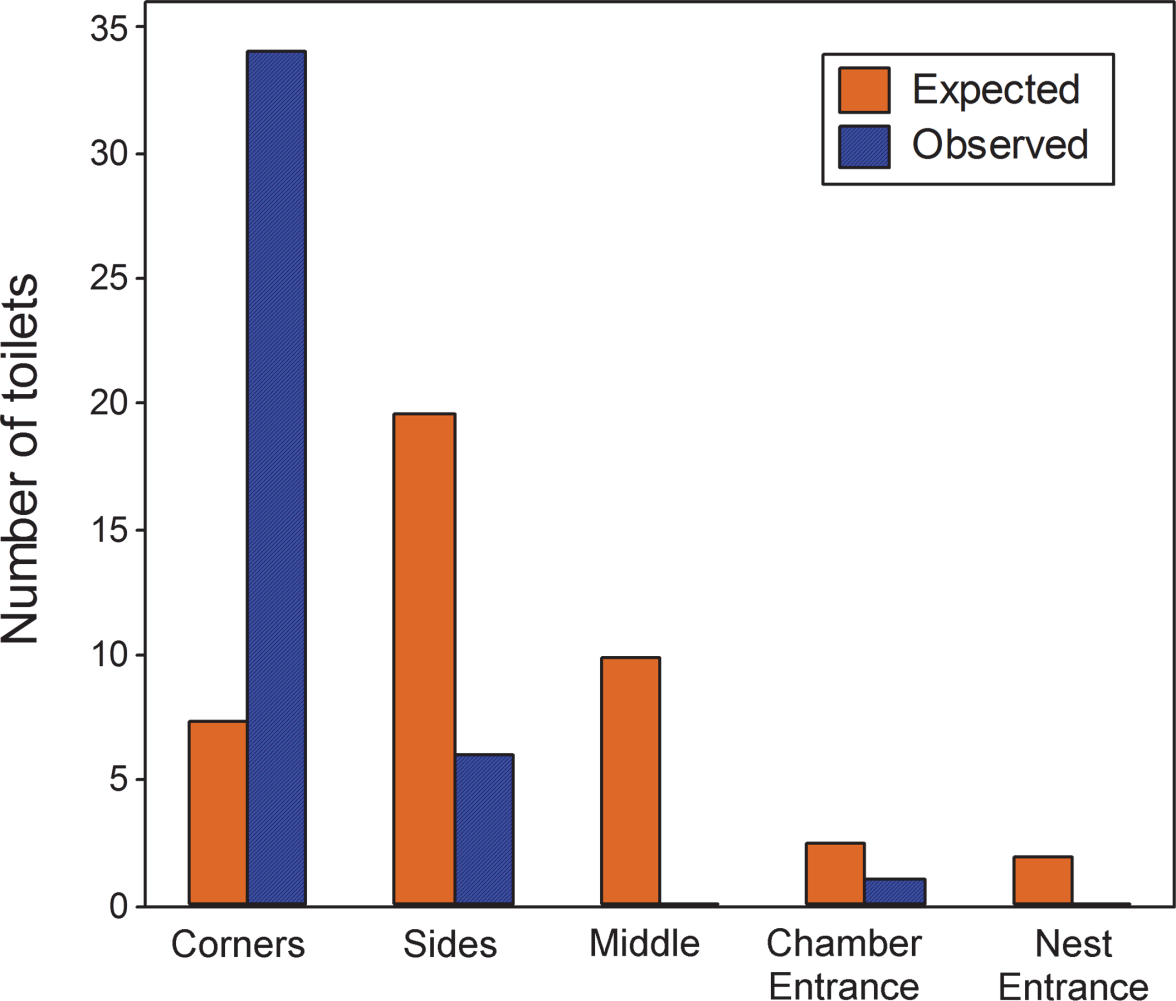
Observed vs expected counts of ant toilets by their location in the nest. Toilets are not randomly located (Χ^2^ test, P < 0.001), but rather are over-represented in the corners of nest chambers (see Fig. 2).

The patches were not randomly distributed around the nest, but rather localised primarily in the corners of the chambers (34 / 41 patches, Chi-squared value 118.67, P < 0.001, Fig. 3).

The patches never contained debris, dead ants, or other solid waste materials from the nest. However, distinct piles of waste debris were formed outside the nest in all 21 colonies. These piles contained dead ants, nest debris (e.g. cotton wool from the water plugs, small pieces of plaster), and coloured particles of the protein food source. In three of the 21 colonies a distinct patch of the same colour as the sugar solution was also found outside the nest, in addition to the patch/patches found inside the nest. The ants may have considered some parts of their foraging arena as part of their nest. Apart from their colouration, the patches were very similar to those observed to form in ants nests kept for other experimental reasons. Pictures of several such nests are provided in S2 Images. A dark, mud-like substance could be observed in the centre of the more developed patches in both this experiment and in uncoloured patches found in other nests.

While pictures of the nest and foraging arena were taken every week (available from Dryad, doi:10.5061/dryad.9fs7n), it was not possible to track the formation of the toilets in detail, as the ants often clustered on the location of the toilet. We chose not to remove the ants from the colony every week, so as to minimise disturbance. Likewise, the location of brood piles could not always be clearly discerned.

## Discussion

The colour of the dark patches in the nests matched the colour of the sucrose solution presented to the ants. The presence of distinct coloured patches in the ant nests thus strongly suggests that the ants were defecating in a specific location in the nest. These patches were distinct from previously reported waste chambers, refuse heaps, or kitchen middens [7–9] in that they never contained food waste, nest debris, or any other solid items. We thus feel justified in terming these patches ‘toilets’. We have observed similar patches in nests of experimental colonies used for other purposes (S2 Images). Such toilets are formed even when there are many unused chambers in the nest. Thus, these toilets are also not simply the result of overcrowding or the design of the experimental nests used in this study. While we could not directly observe defecation in this study, the appearance of the toilets is not consistent with other potential explanations. For example, the presence of a mud-like substance in the toilets precludes failed trophallaxis of the sugar water, as does the tight localisation of the patches. The lack of any protein colour in the toilets was presumably because the ants consumed only very small amounts of the protein source.

This study is, to our knowledge, the first formal description of ants forming distinct toilets. However, similar structures may have been observed in *Crematogaster smithi* (S. Cremer & J. Oettler, Pers. Com), suggesting that this behaviour is not an idiosyncrasy of *Lasius niger*. An intriguing question arising from this finding is why the ants go to the effort of forming such a toilet. Intuitively, the answer seems likely to be connected to sanitary behaviour: faeces is normally assumed to be a source of dangerous pathogens, and can be a source of dangerous pathogens in honeybees and other insects [1,2], and so localising it in one location in the nest seems sensible.

However, the ants in this study did not seem to avoid the location of the toilets (although the location of brood was usually not possible to ascertain, see S1 Images). Moreover, an apparently more sensible solution would be to simply defecate outside the nest, as honey bees do [4]. It may be that ants, which cannot fly, would have to travel much further to deposit faeces outside of well-visited areas. Another possibility is that the young or inactive ants form these toilets to avoid leaving the nest at all; larval faeces is collected in a meconium attached to the cocoon, and is expelled from the nest to refuse piles after emergence of the adult ants. However, young honey bees are known to leave the nest on defecation flights, so why inactive ants should not do so is unclear. Perhaps walking on the ground is more dangerous than flying. An alternative solution would be one used by some stingless bee species, in which faeces is collected in piles and regularly removed by specialised workers [23]. However, the faeces produced by *L*. *niger* may be liquid, and so not easily transportable. A second possibility is that ants avoid defecating outside the nest so as to avoid attracting predators to the nest, which is, for example, the reason many frass-flinging caterpillars remove their faeces [24]. However, as *L*. *niger* form pheromone trails leading directly to the nest, mark the nest surroundings with home-range markings, and remove corpses and food waste [25,26], this explanation also seems unlikely.

While it is usually assumed that behaviours dealing with faeces arise for sanitary reasons, an alternative possibility is that the faeces is not harmful [24]. It is telling that, in contrast to faeces, dead nestmates—which do pose a risk to the colony—are removed from the nest [26]. Indeed, the toilets may provide a useful function. Leaf cutter ants manure their fungal gardens—their food source—by defecating on them. Similarly, fungus-growing termites construct their fungus growing substrate from partially digested faeces [27]. Many termites construct their nest partially or entirely using their own faeces [19]. *Oecophylla longinoda* ants mark their home-range and foraging trails with faecal markings [28]. This suggests that faeces are not always a source of dangerous infectious material, are not necessarily avoided, and may indeed be beneficial. The toilets formed by *L*. *niger* may also have a beneficial role. For example, they may act as a source of salt or micronutrients. The nutritional requirements of adults and larvae are no doubt very different, and it is conceivable that nutrients ingested but not used by the adults would accumulate in the toilets, and then be fed to the larvae. A second possibility is that the faeces may have an antimicrobial effect, as reported in some termites [20]. However, pilot experiments on the toilets of *C*. *smithi* ants revealed no evidence of anti-fungal activity of their faeces (S. Cremer, pers. comm.). Moreover, we have observed that, in nests devoid of ants, fungus-like fruiting bodies begin to grow on the toilet patches. The growth of micro-organisms on the toilets may be actively inhibited by the ants, perhaps by the application of formic acid or antibiotic secretions [13,15]. Alternatively, the ants may be using the toilets as a garden, and eating the resulting fruiting bodies, as a way of accessing otherwise inaccessible nutrients from their waste. These hypothesised roles for the toilets are open to future investigation.

The locations of the toilets were not random, with most being formed in the corner of a chamber. This may be in order to avoid forcing ants to walk through the toilets, although the ants did not seem to avoid the toilets (see S1 Images). Alternatively, corners may simply provide a practical feature to act as a nucleation point for stygmergic behaviour—where the way in which one individual changes the environment guides the future behaviour of its fellows [29]. We have observed that the ponerine ant *Platythyrea punctata* form combined faeces and waste piles preferentially in the corners of their nests when provided with rectangular cavities (T. Czaczkes and A. Bernadou, pers. obs., pictures available upon request). As *L*. *niger* ants tend to aggregate in tight clusters inside the nest, it is possible that the location of the toilet is simply an epiphenomenon of where the ants choose to cluster. However, the ant clusters are often much larger than the tightly localised toilets, strongly suggesting that ants do actively search out the toilet to defecate.

Collective organisation in social insects has been extensively studied in terms of nest construction [27], foraging [30], and defence [31,32]. However, the organisation of refuse management has generally been neglected. Here we show that a common ant, *L*. *niger*, forms well-defined toilets, and outline an easy and effective method for the visualisation of these toilets. Many further questions await investigation, however: are brood brought to, or kept from, the toilets? Do the toilets form via stygmergic mechanisms? Do the toilets have anti-microbial activities, or act as a source of dangerous pathogens? How widespread is this behaviour? Our description of these ant toilets is a first step in understanding these distinct nest structures. Their formation, characteristics, and role are now subjects ripe for study.

## Supporting Information

S1 Images Final state of nests with ants. Pictures of the final state of each nest and nest-box, before the ants were removed from the nest. The images were taken just before the ants were removed from the nest boxes and the photographs in Fig. 2 were taken. The dot and letter in Fig. 1 and 2 represent the “front” of the nest—i.e. the side of the nest furthest from the foraging arena wall. The opaque nest cover was removed only seconds before the photographs were taken. Similar photographs taken every week throughout the course of the experiment are available from Dryad (doi:10.5061/dryad.9fs7n).
(ZIP)

S2 Images Uncoloured ant toilets. We maintain larger *Lasius niger* colonies in our lab in plaster nests similar to those described in this study. The ants are periodically rehoused or discarded. This supplement contains a selection of images of such plaster nests, with occasional close-up pictures of the toilets.
(ZIP)

S1 Methods Detailed methods description. Detailed methods including details of ant maintenance, food and nest preparation, data collections, and the instructions given to the blind observer.
(PDF)
