# Supplementary figures and images for: Nest Etiquette—Where Ants Go When Nature Calls
Source: PLoS One. 2015 Feb 18;10(2):e0118376. doi: 10.1371/journal.pone.0118376 (PMC4332866; doi:10.1371/journal.pone.0118376)

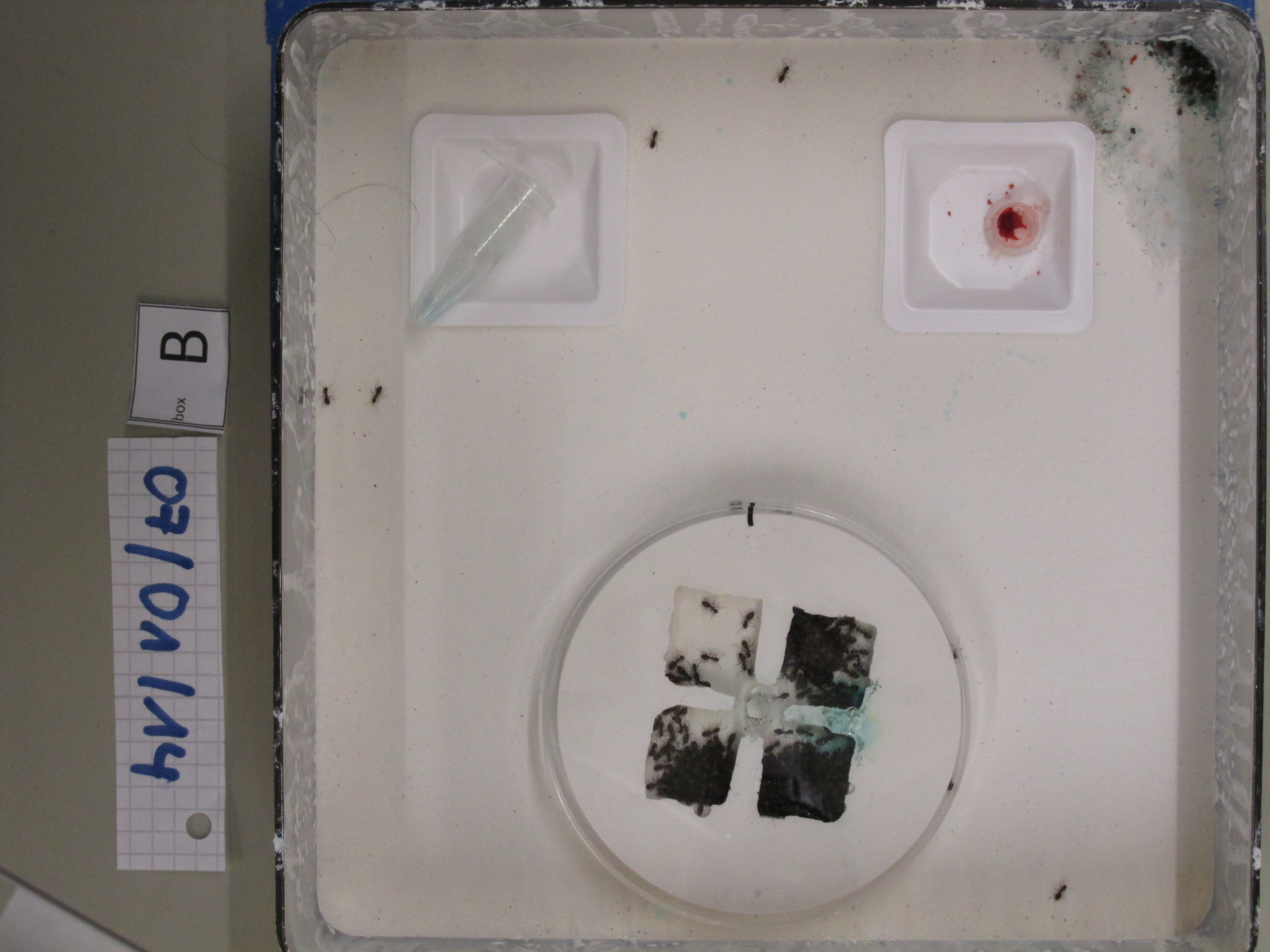

Supplement: S1 Images — Pictures of the final state of each nest and nest-box, before the ants were removed from the nest. The images were taken just before the ants were removed from the nest boxes and the photographs in Fig. 2 were taken. The dot and letter in Fig. 1 and 2 represent the “front” of the nest—i.e. the side of the nest furthest from the foraging arena wall. The opaque nest cover was removed only seconds before the photographs were taken. Similar photographs taken every week throughout the course of the experiment are available from Dryad (doi:10.5061/dryad.9fs7n). (ZIP) [file pone.0118376.s001.zip › BO 070114 (2).JPG]

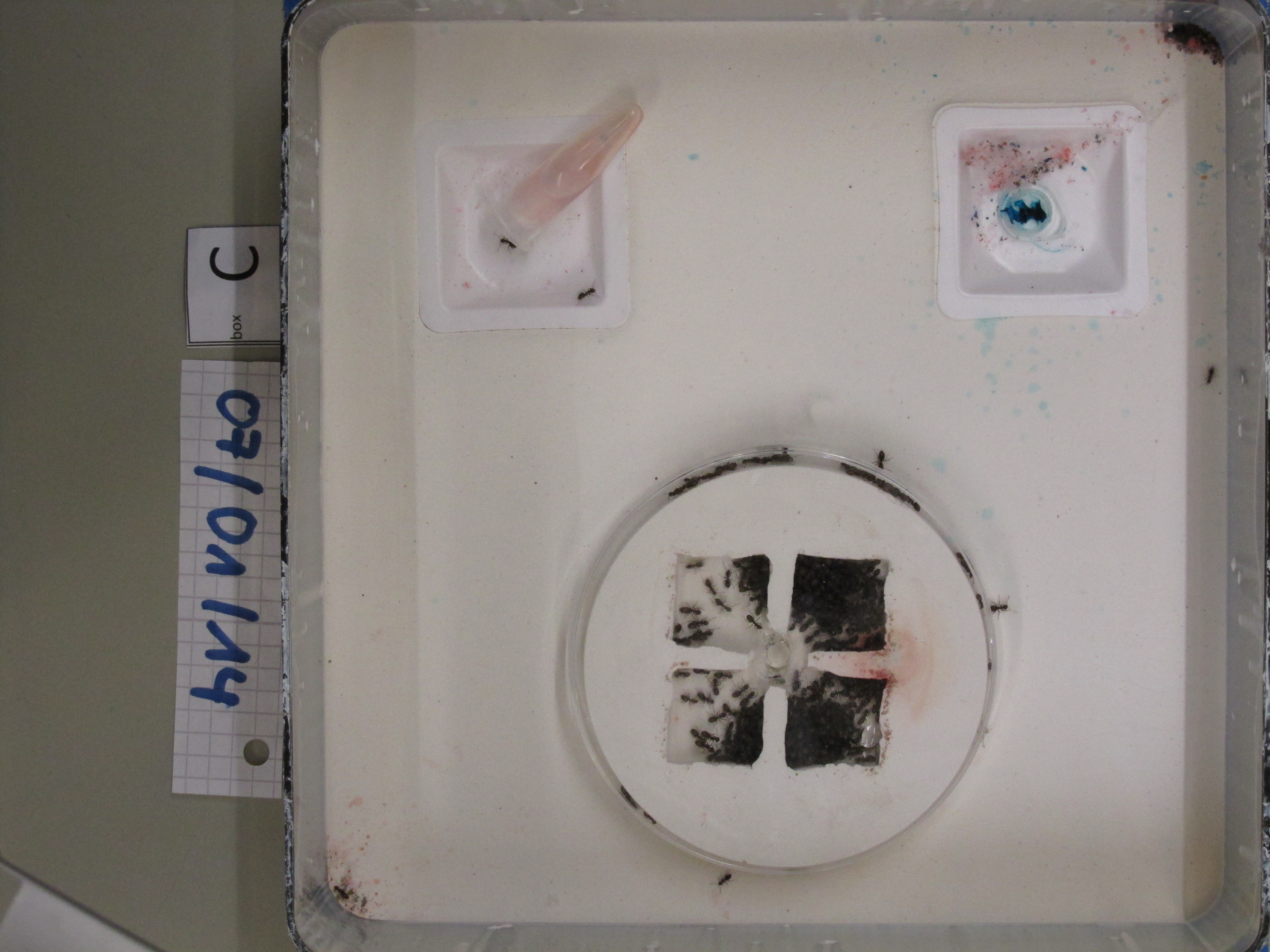

Supplement: S1 Images — Pictures of the final state of each nest and nest-box, before the ants were removed from the nest. The images were taken just before the ants were removed from the nest boxes and the photographs in Fig. 2 were taken. The dot and letter in Fig. 1 and 2 represent the “front” of the nest—i.e. the side of the nest furthest from the foraging arena wall. The opaque nest cover was removed only seconds before the photographs were taken. Similar photographs taken every week throughout the course of the experiment are available from Dryad (doi:10.5061/dryad.9fs7n). (ZIP) [file pone.0118376.s001.zip › CO 070114 (38).JPG]

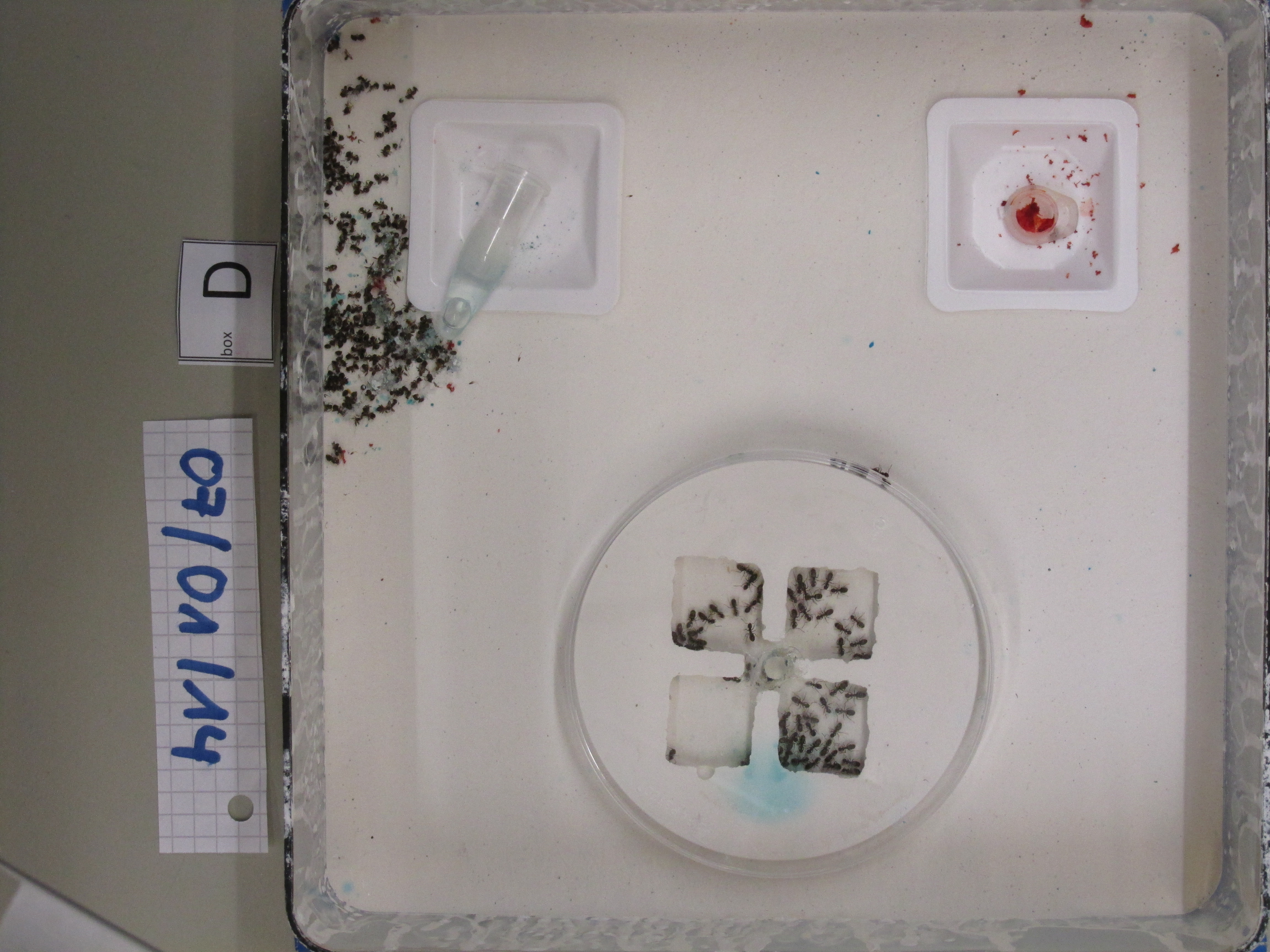

Supplement: S1 Images — Pictures of the final state of each nest and nest-box, before the ants were removed from the nest. The images were taken just before the ants were removed from the nest boxes and the photographs in Fig. 2 were taken. The dot and letter in Fig. 1 and 2 represent the “front” of the nest—i.e. the side of the nest furthest from the foraging arena wall. The opaque nest cover was removed only seconds before the photographs were taken. Similar photographs taken every week throughout the course of the experiment are available from Dryad (doi:10.5061/dryad.9fs7n). (ZIP) [file pone.0118376.s001.zip › DO 070114 (18).JPG]

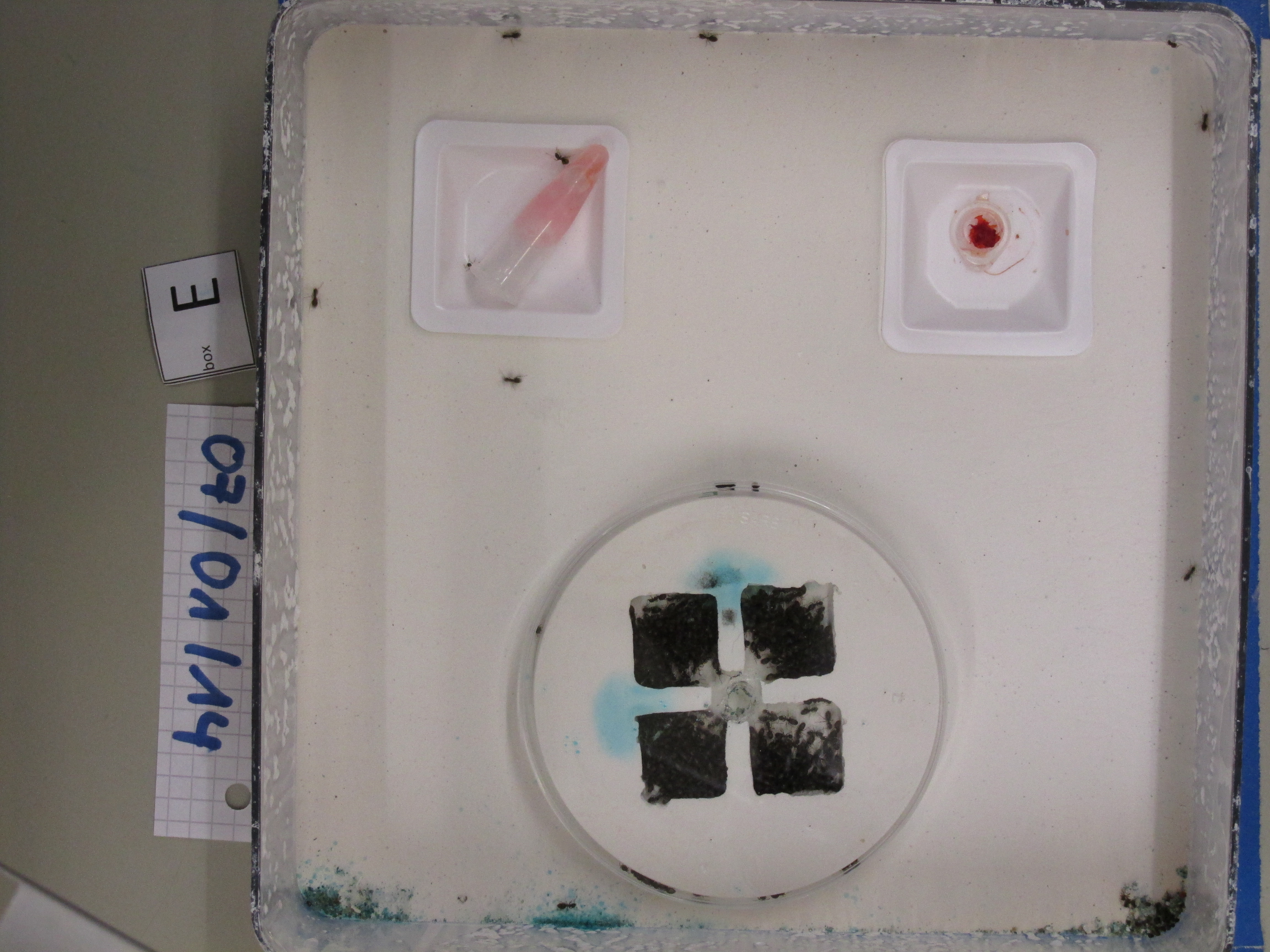

Supplement: S1 Images — Pictures of the final state of each nest and nest-box, before the ants were removed from the nest. The images were taken just before the ants were removed from the nest boxes and the photographs in Fig. 2 were taken. The dot and letter in Fig. 1 and 2 represent the “front” of the nest—i.e. the side of the nest furthest from the foraging arena wall. The opaque nest cover was removed only seconds before the photographs were taken. Similar photographs taken every week throughout the course of the experiment are available from Dryad (doi:10.5061/dryad.9fs7n). (ZIP) [file pone.0118376.s001.zip › EO 070114 (16).JPG]

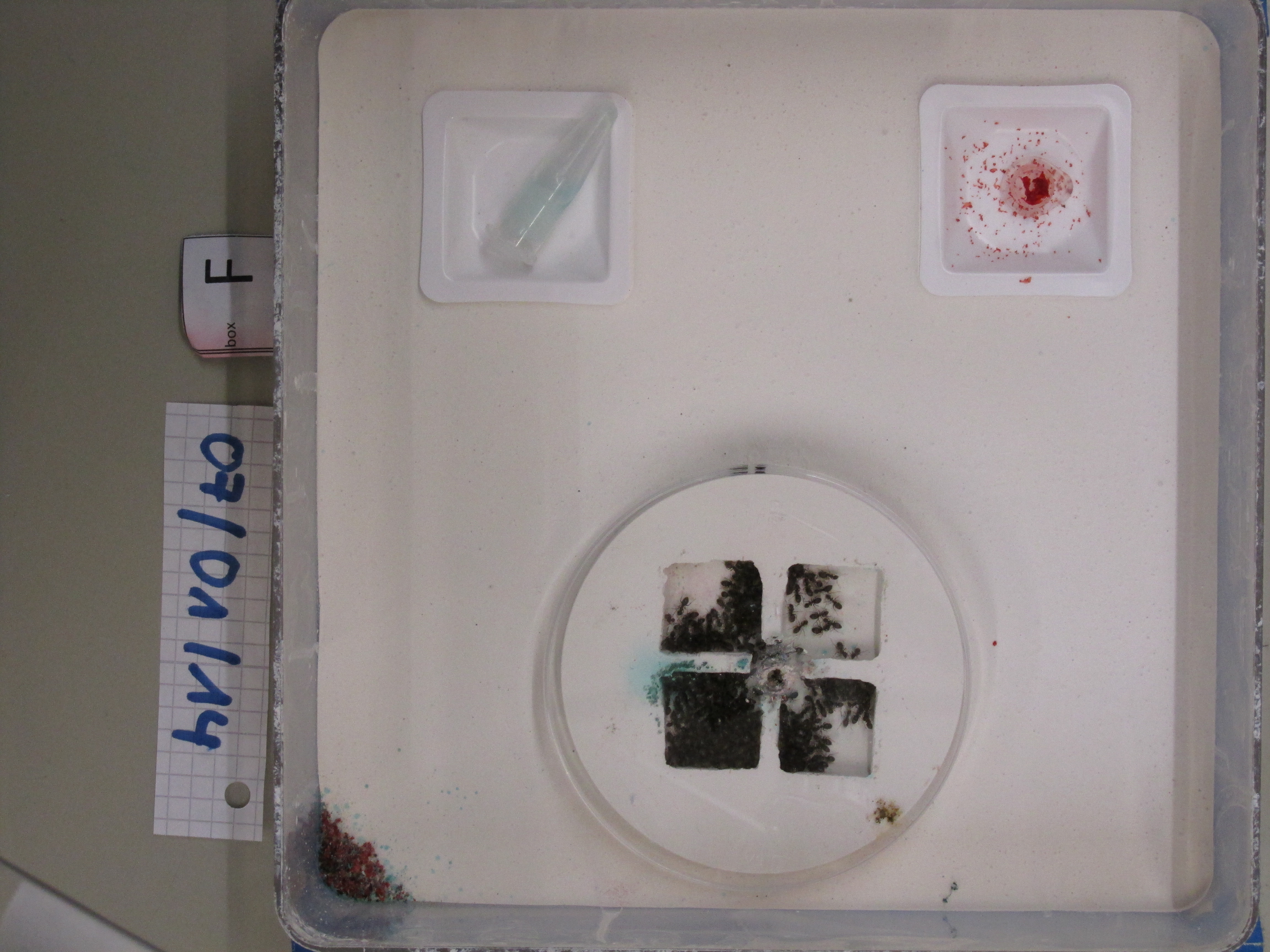

Supplement: S1 Images — Pictures of the final state of each nest and nest-box, before the ants were removed from the nest. The images were taken just before the ants were removed from the nest boxes and the photographs in Fig. 2 were taken. The dot and letter in Fig. 1 and 2 represent the “front” of the nest—i.e. the side of the nest furthest from the foraging arena wall. The opaque nest cover was removed only seconds before the photographs were taken. Similar photographs taken every week throughout the course of the experiment are available from Dryad (doi:10.5061/dryad.9fs7n). (ZIP) [file pone.0118376.s001.zip › FO 070114 (10).JPG]

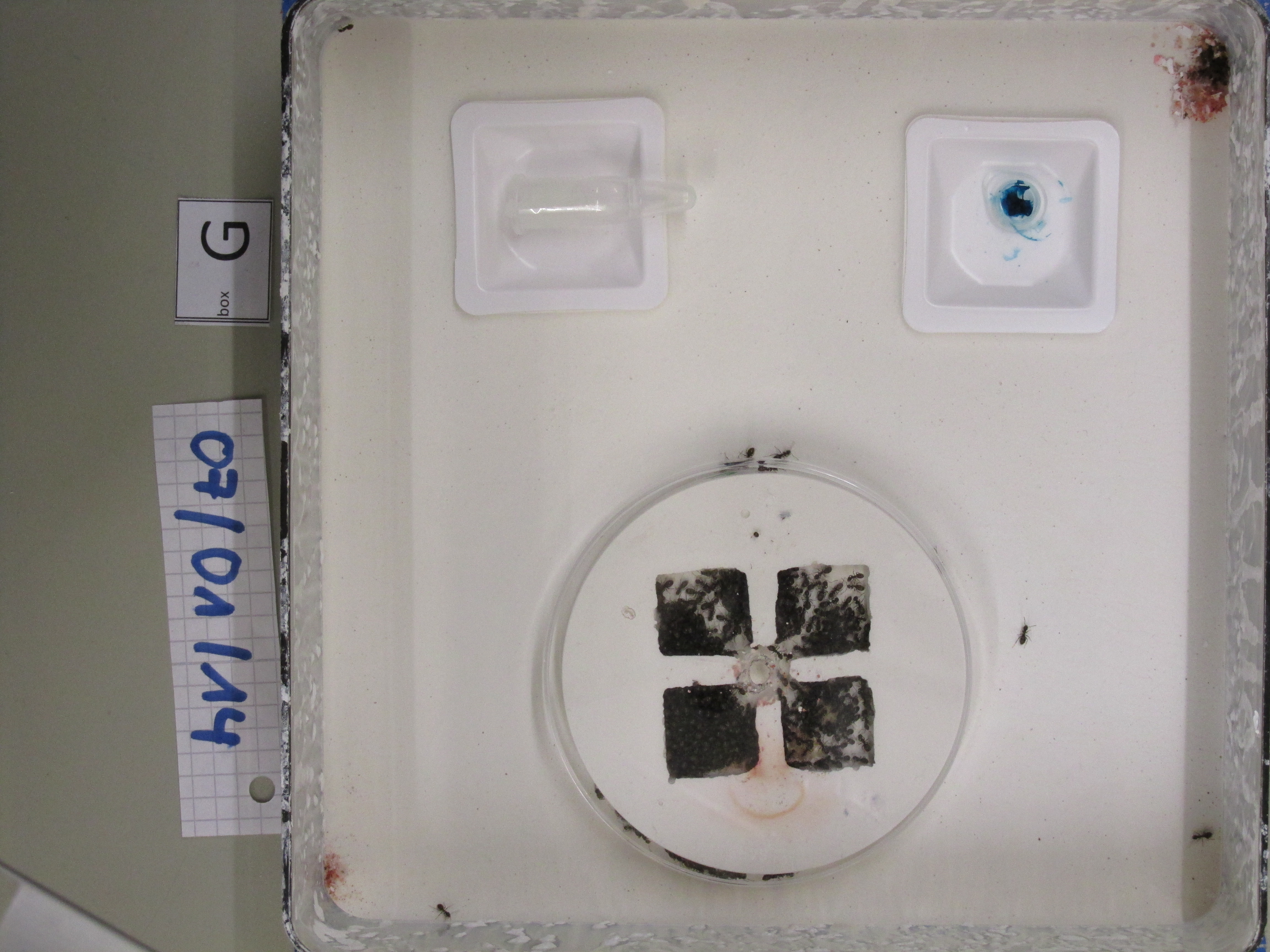

Supplement: S1 Images — Pictures of the final state of each nest and nest-box, before the ants were removed from the nest. The images were taken just before the ants were removed from the nest boxes and the photographs in Fig. 2 were taken. The dot and letter in Fig. 1 and 2 represent the “front” of the nest—i.e. the side of the nest furthest from the foraging arena wall. The opaque nest cover was removed only seconds before the photographs were taken. Similar photographs taken every week throughout the course of the experiment are available from Dryad (doi:10.5061/dryad.9fs7n). (ZIP) [file pone.0118376.s001.zip › GO 070114 (24).JPG]

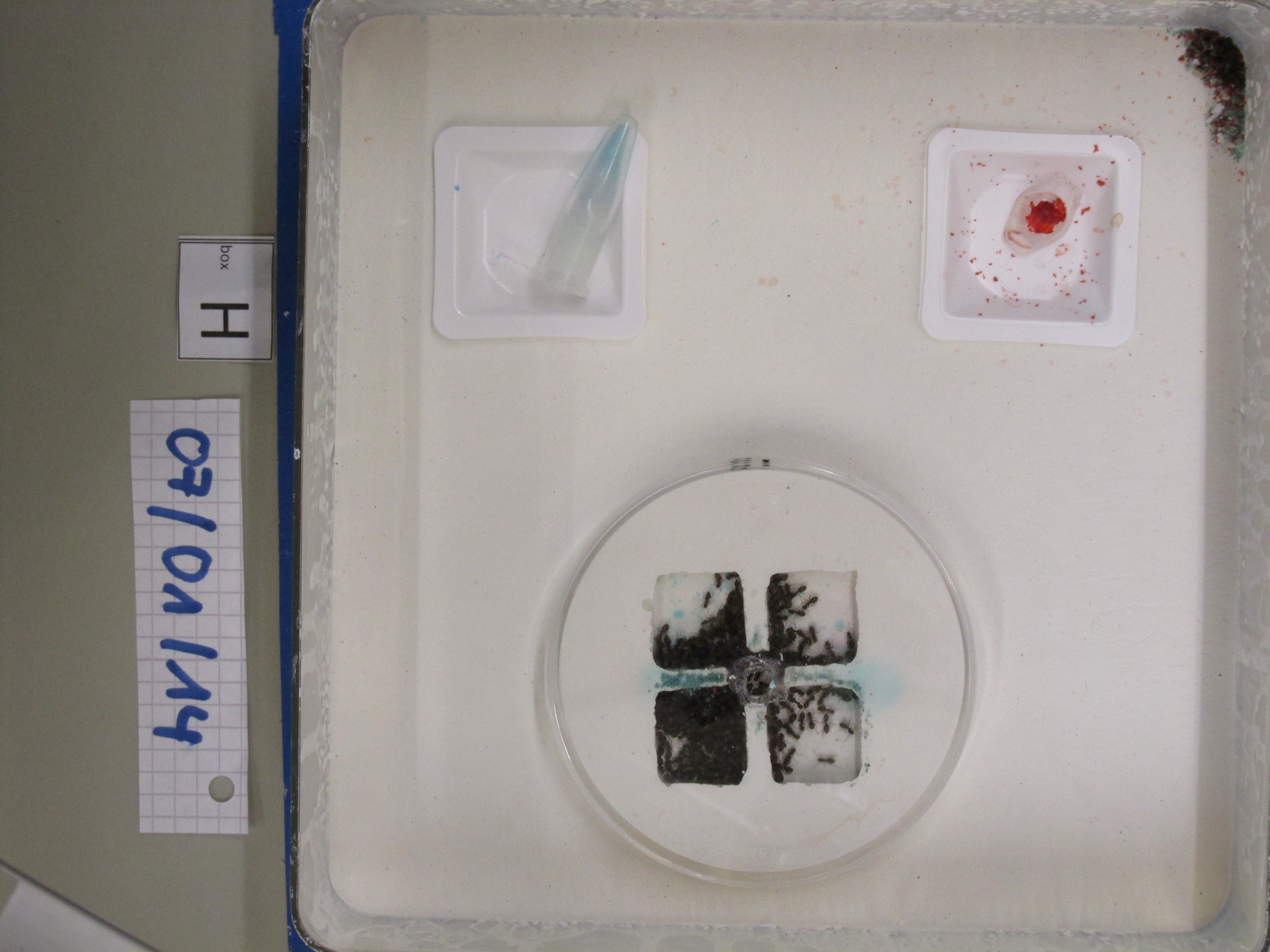

Supplement: S1 Images — Pictures of the final state of each nest and nest-box, before the ants were removed from the nest. The images were taken just before the ants were removed from the nest boxes and the photographs in Fig. 2 were taken. The dot and letter in Fig. 1 and 2 represent the “front” of the nest—i.e. the side of the nest furthest from the foraging arena wall. The opaque nest cover was removed only seconds before the photographs were taken. Similar photographs taken every week throughout the course of the experiment are available from Dryad (doi:10.5061/dryad.9fs7n). (ZIP) [file pone.0118376.s001.zip › HO 070114 (6).JPG]

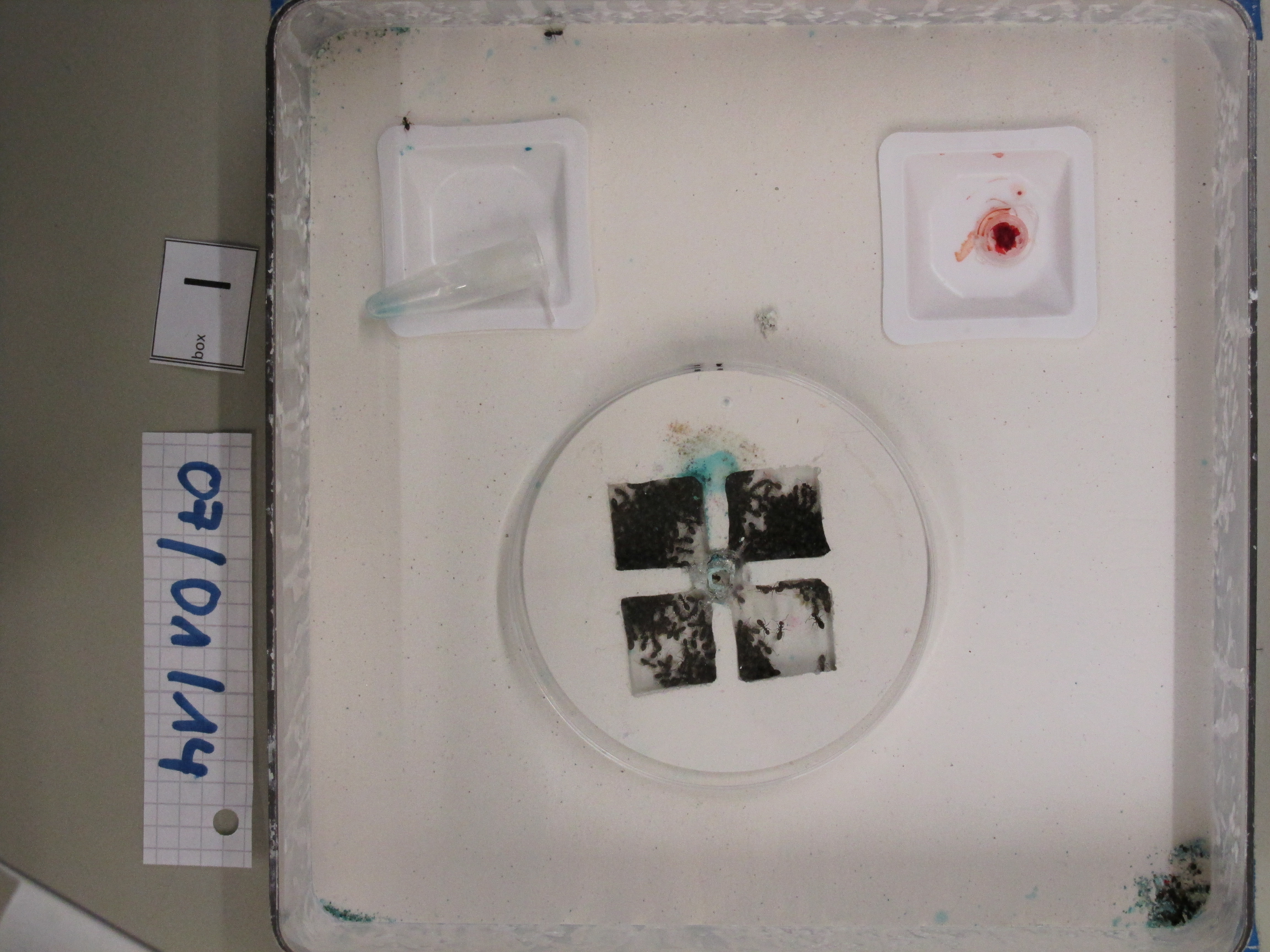

Supplement: S1 Images — Pictures of the final state of each nest and nest-box, before the ants were removed from the nest. The images were taken just before the ants were removed from the nest boxes and the photographs in Fig. 2 were taken. The dot and letter in Fig. 1 and 2 represent the “front” of the nest—i.e. the side of the nest furthest from the foraging arena wall. The opaque nest cover was removed only seconds before the photographs were taken. Similar photographs taken every week throughout the course of the experiment are available from Dryad (doi:10.5061/dryad.9fs7n). (ZIP) [file pone.0118376.s001.zip › IO 070114 (4).JPG]

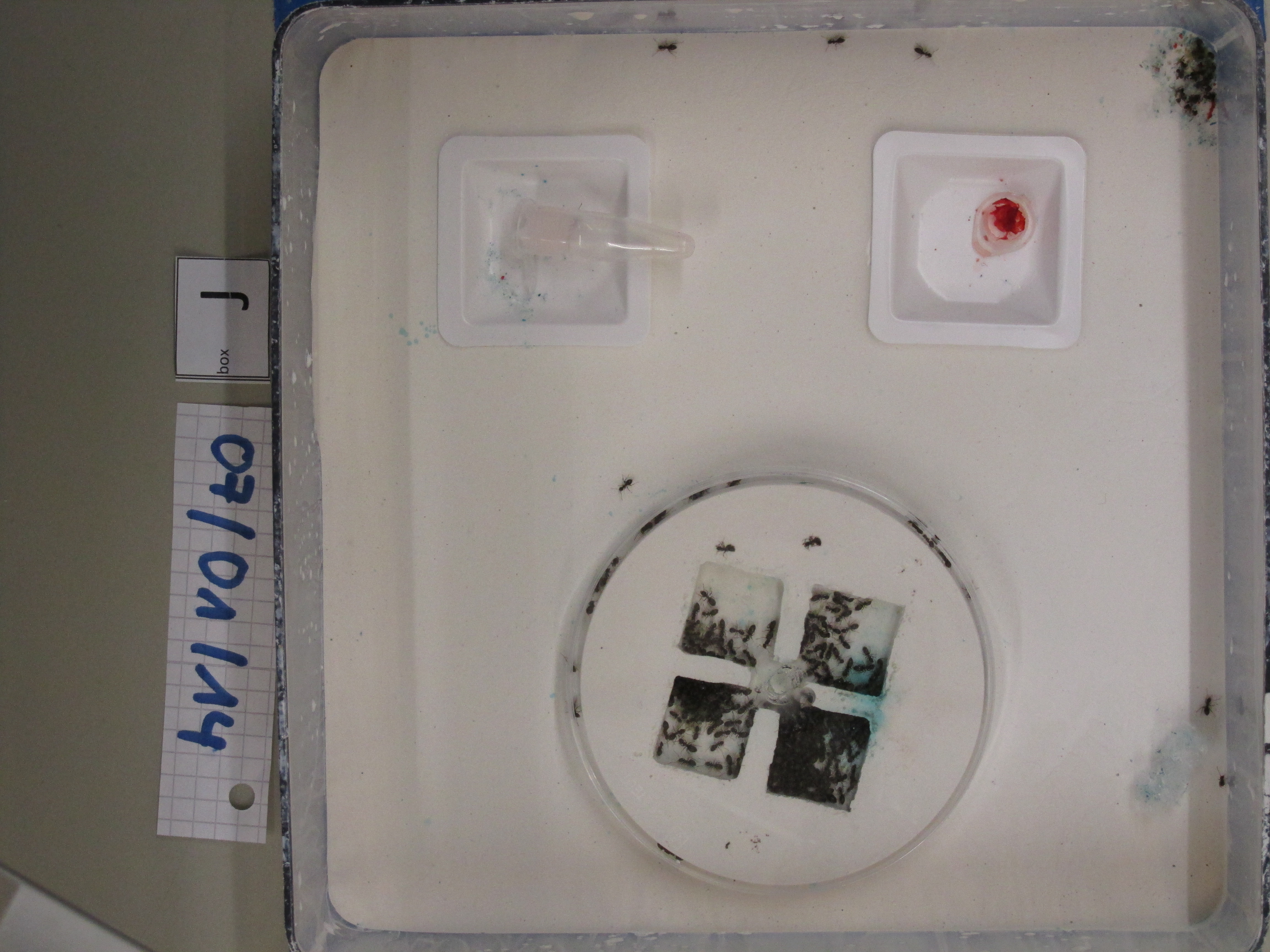

Supplement: S1 Images — Pictures of the final state of each nest and nest-box, before the ants were removed from the nest. The images were taken just before the ants were removed from the nest boxes and the photographs in Fig. 2 were taken. The dot and letter in Fig. 1 and 2 represent the “front” of the nest—i.e. the side of the nest furthest from the foraging arena wall. The opaque nest cover was removed only seconds before the photographs were taken. Similar photographs taken every week throughout the course of the experiment are available from Dryad (doi:10.5061/dryad.9fs7n). (ZIP) [file pone.0118376.s001.zip › JO 070114 (8).JPG]

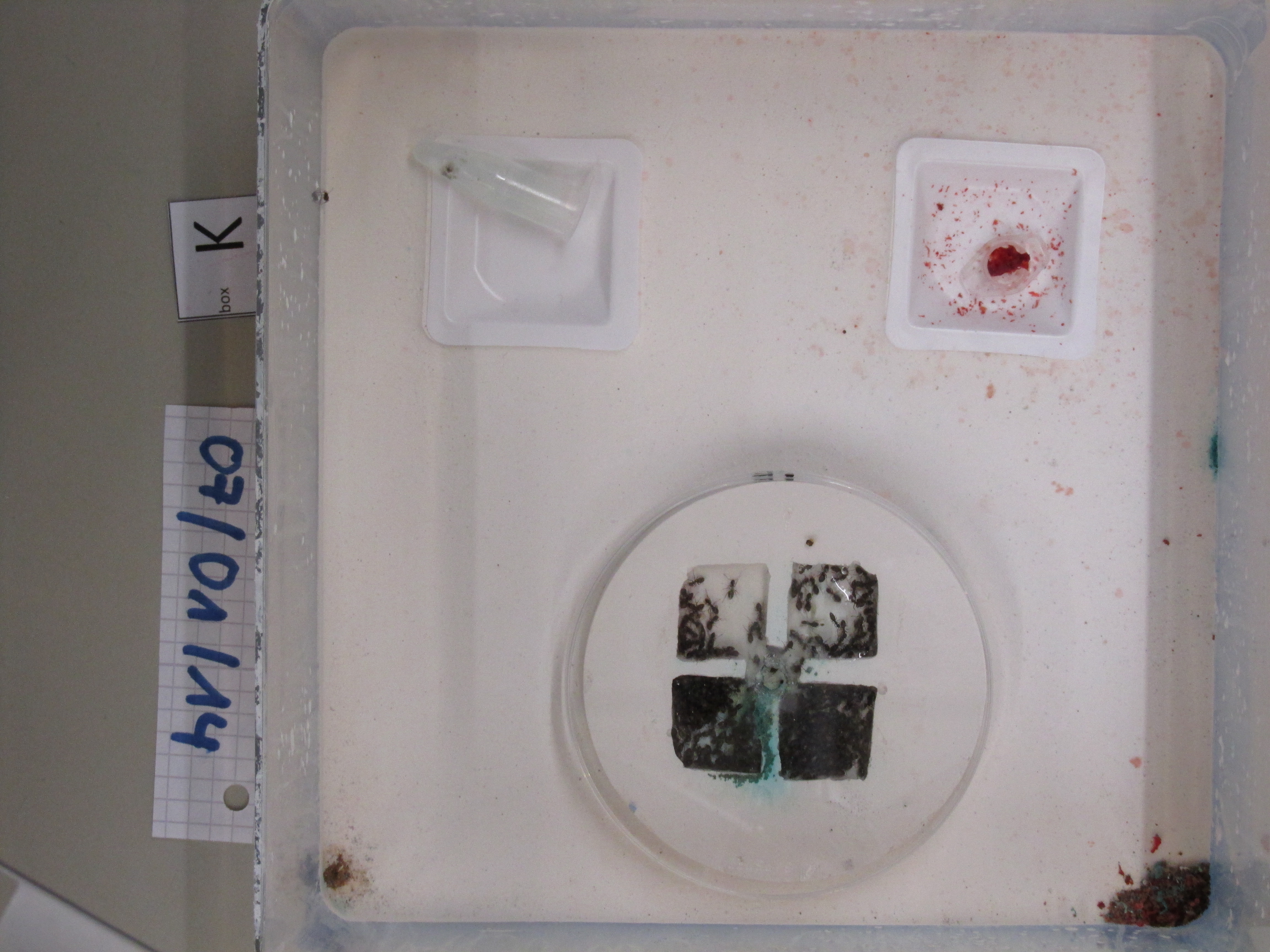

Supplement: S1 Images — Pictures of the final state of each nest and nest-box, before the ants were removed from the nest. The images were taken just before the ants were removed from the nest boxes and the photographs in Fig. 2 were taken. The dot and letter in Fig. 1 and 2 represent the “front” of the nest—i.e. the side of the nest furthest from the foraging arena wall. The opaque nest cover was removed only seconds before the photographs were taken. Similar photographs taken every week throughout the course of the experiment are available from Dryad (doi:10.5061/dryad.9fs7n). (ZIP) [file pone.0118376.s001.zip › KO 070114 (12).JPG]

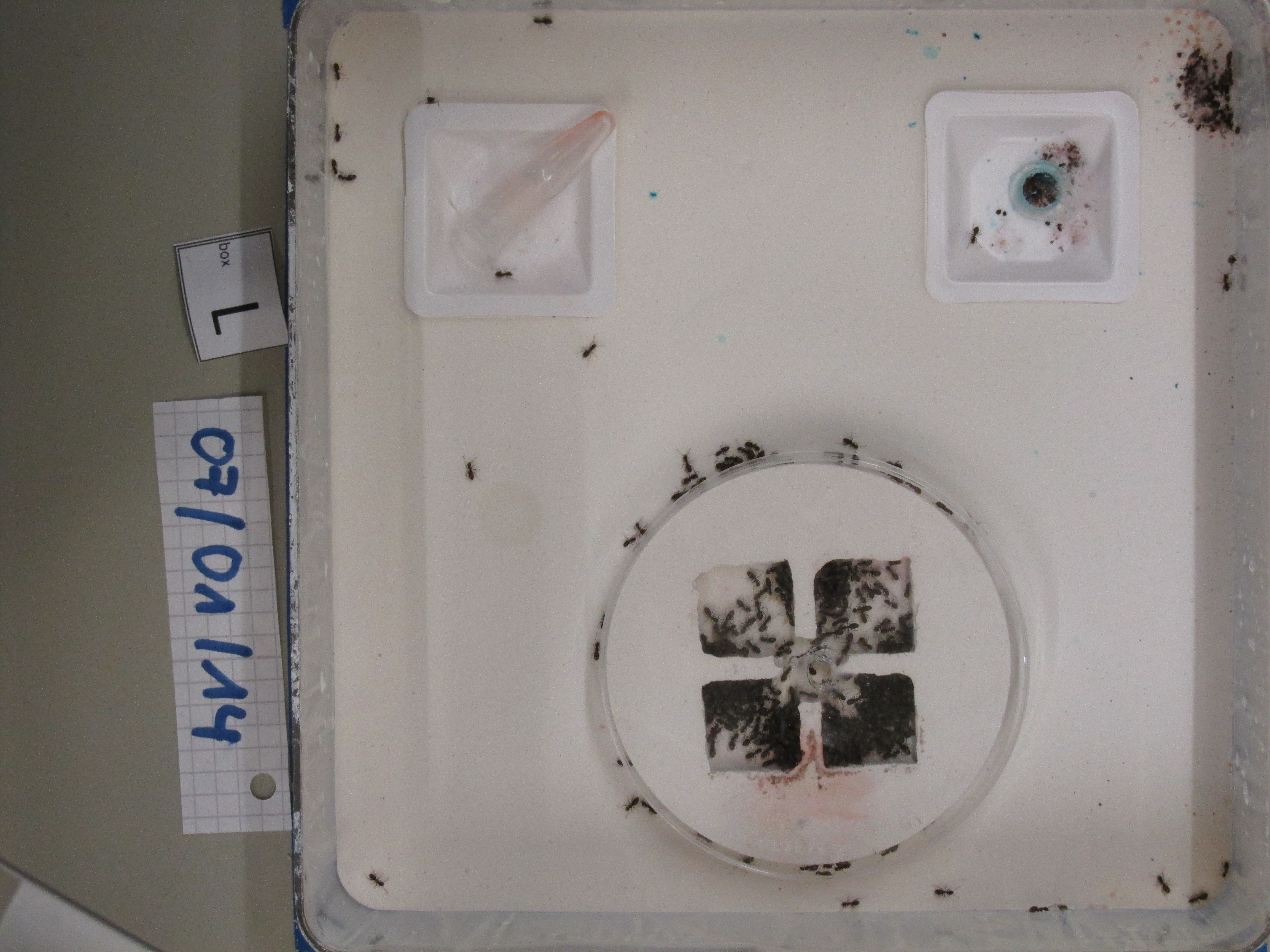

Supplement: S1 Images — Pictures of the final state of each nest and nest-box, before the ants were removed from the nest. The images were taken just before the ants were removed from the nest boxes and the photographs in Fig. 2 were taken. The dot and letter in Fig. 1 and 2 represent the “front” of the nest—i.e. the side of the nest furthest from the foraging arena wall. The opaque nest cover was removed only seconds before the photographs were taken. Similar photographs taken every week throughout the course of the experiment are available from Dryad (doi:10.5061/dryad.9fs7n). (ZIP) [file pone.0118376.s001.zip › LO 070114 (22).JPG]

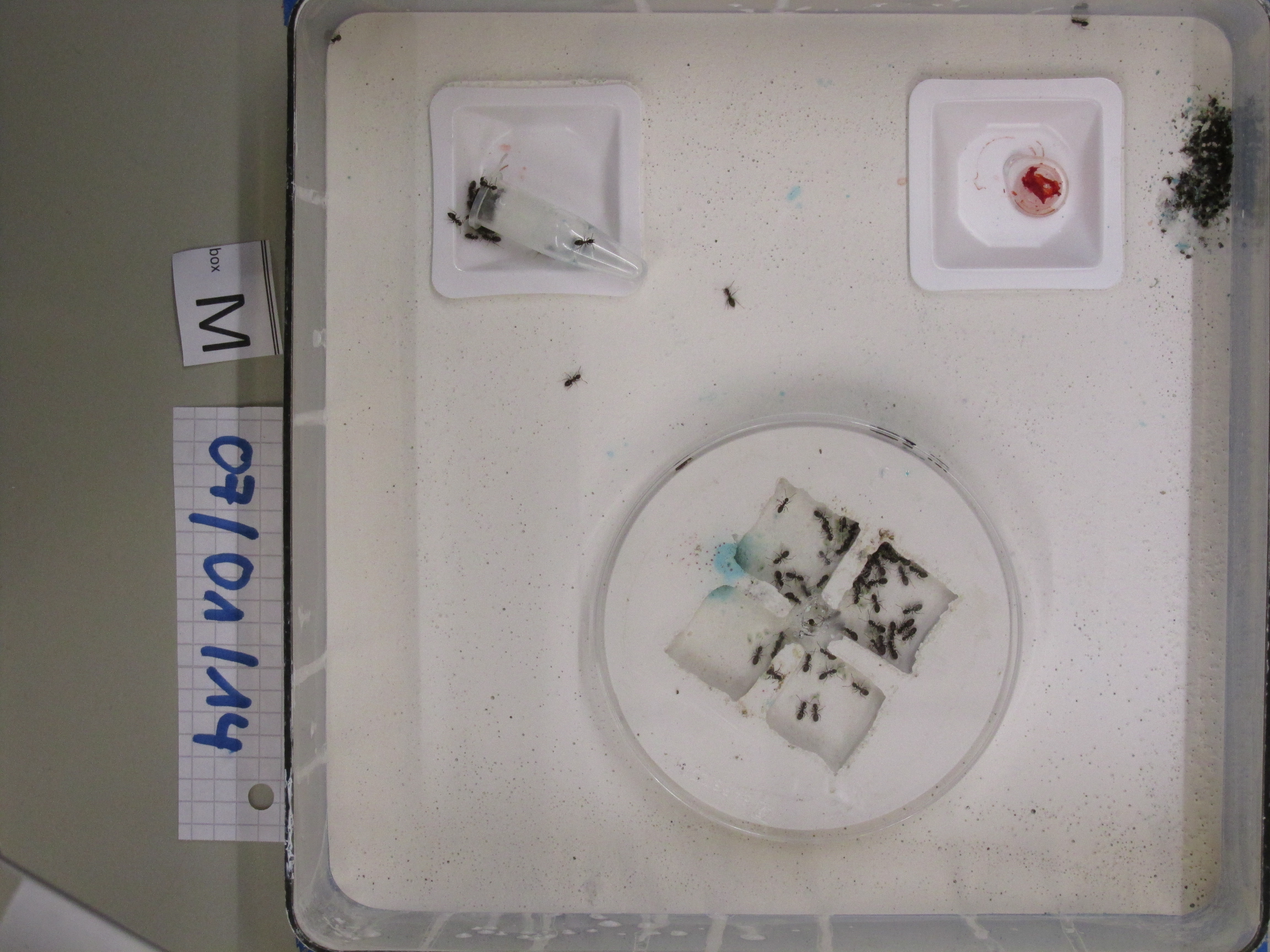

Supplement: S1 Images — Pictures of the final state of each nest and nest-box, before the ants were removed from the nest. The images were taken just before the ants were removed from the nest boxes and the photographs in Fig. 2 were taken. The dot and letter in Fig. 1 and 2 represent the “front” of the nest—i.e. the side of the nest furthest from the foraging arena wall. The opaque nest cover was removed only seconds before the photographs were taken. Similar photographs taken every week throughout the course of the experiment are available from Dryad (doi:10.5061/dryad.9fs7n). (ZIP) [file pone.0118376.s001.zip › MO 070114 (20).JPG]

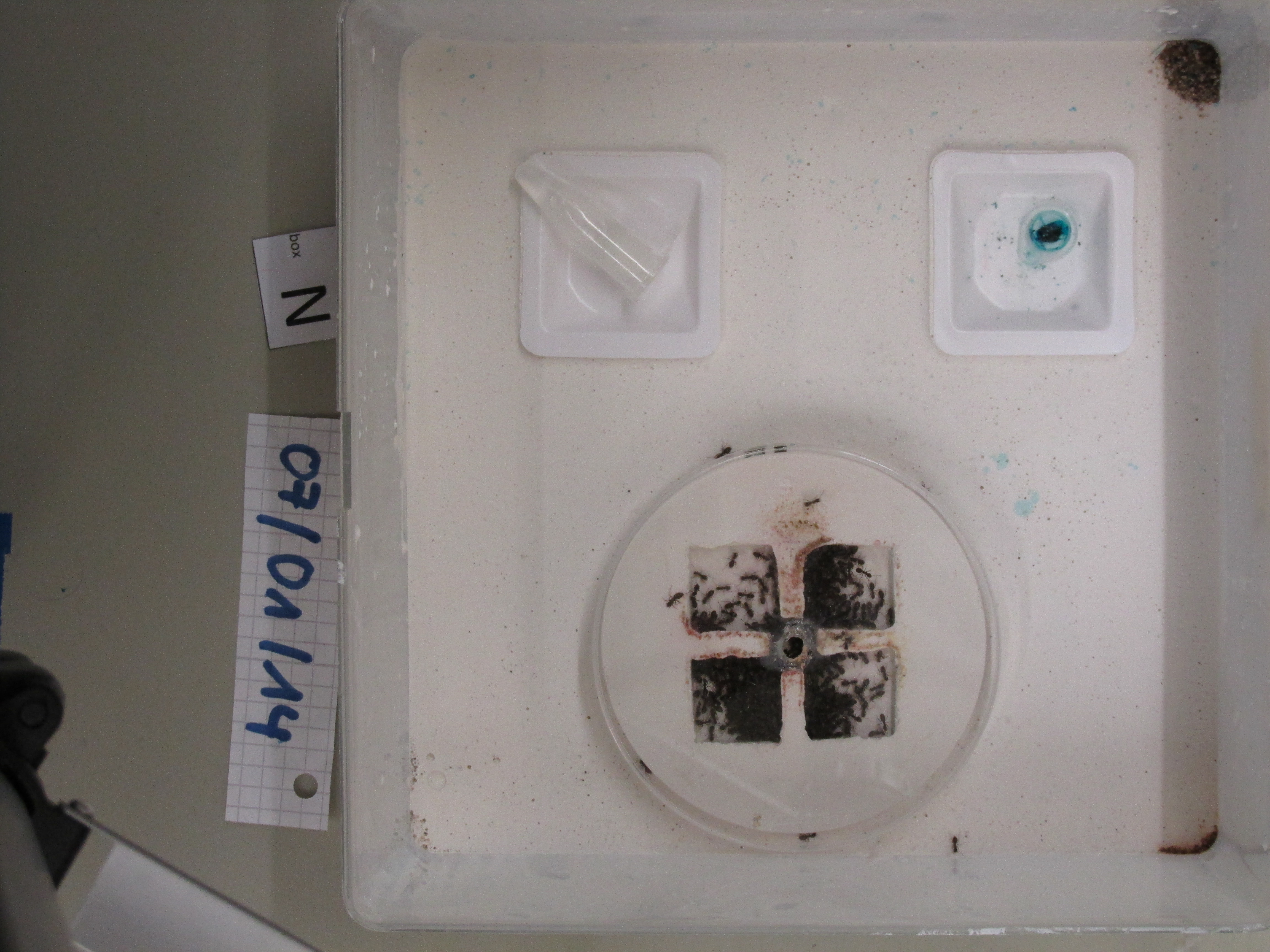

Supplement: S1 Images — Pictures of the final state of each nest and nest-box, before the ants were removed from the nest. The images were taken just before the ants were removed from the nest boxes and the photographs in Fig. 2 were taken. The dot and letter in Fig. 1 and 2 represent the “front” of the nest—i.e. the side of the nest furthest from the foraging arena wall. The opaque nest cover was removed only seconds before the photographs were taken. Similar photographs taken every week throughout the course of the experiment are available from Dryad (doi:10.5061/dryad.9fs7n). (ZIP) [file pone.0118376.s001.zip › NO 070114 (42).JPG]

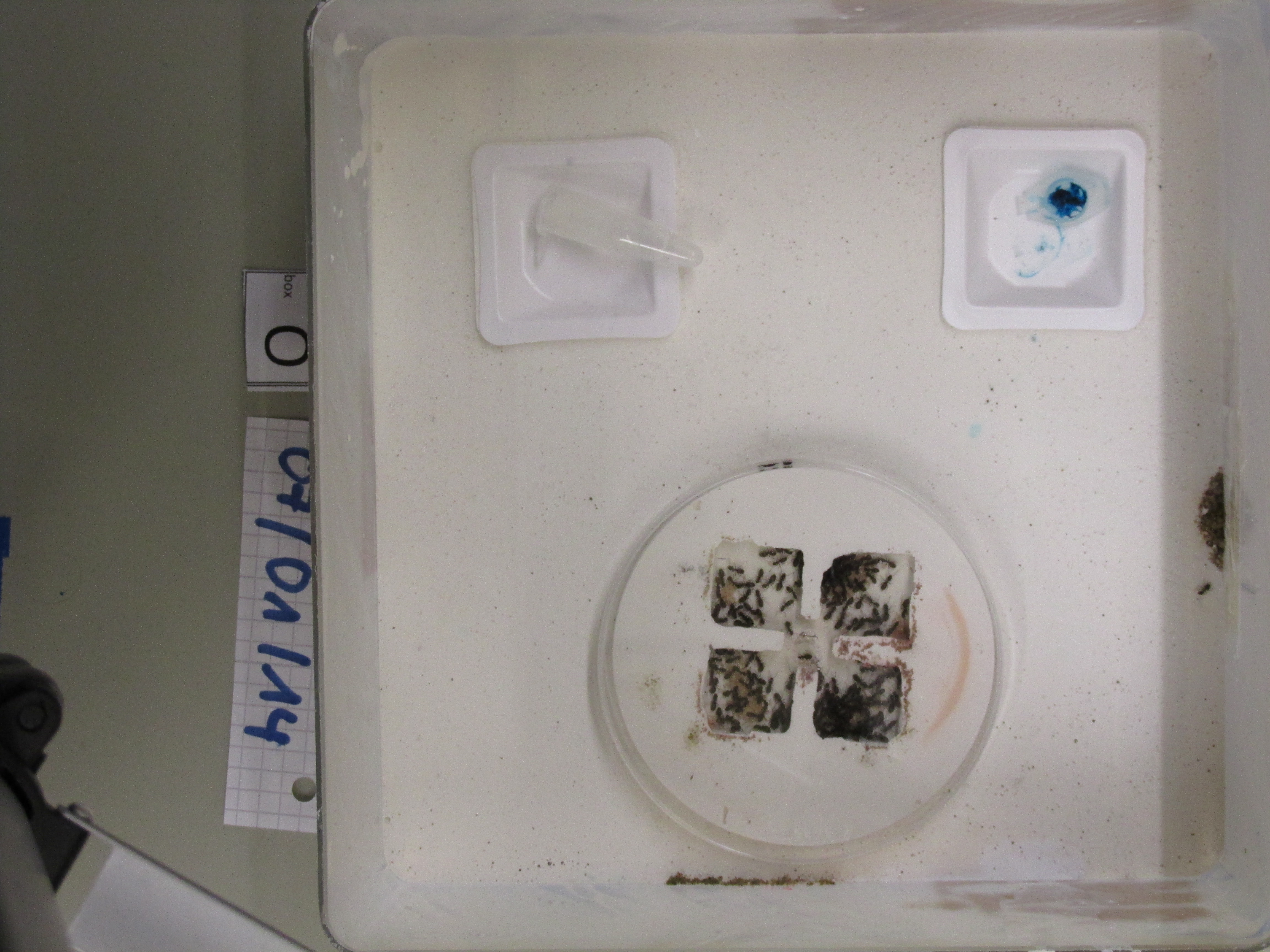

Supplement: S1 Images — Pictures of the final state of each nest and nest-box, before the ants were removed from the nest. The images were taken just before the ants were removed from the nest boxes and the photographs in Fig. 2 were taken. The dot and letter in Fig. 1 and 2 represent the “front” of the nest—i.e. the side of the nest furthest from the foraging arena wall. The opaque nest cover was removed only seconds before the photographs were taken. Similar photographs taken every week throughout the course of the experiment are available from Dryad (doi:10.5061/dryad.9fs7n). (ZIP) [file pone.0118376.s001.zip › OO 070114 (40).JPG]

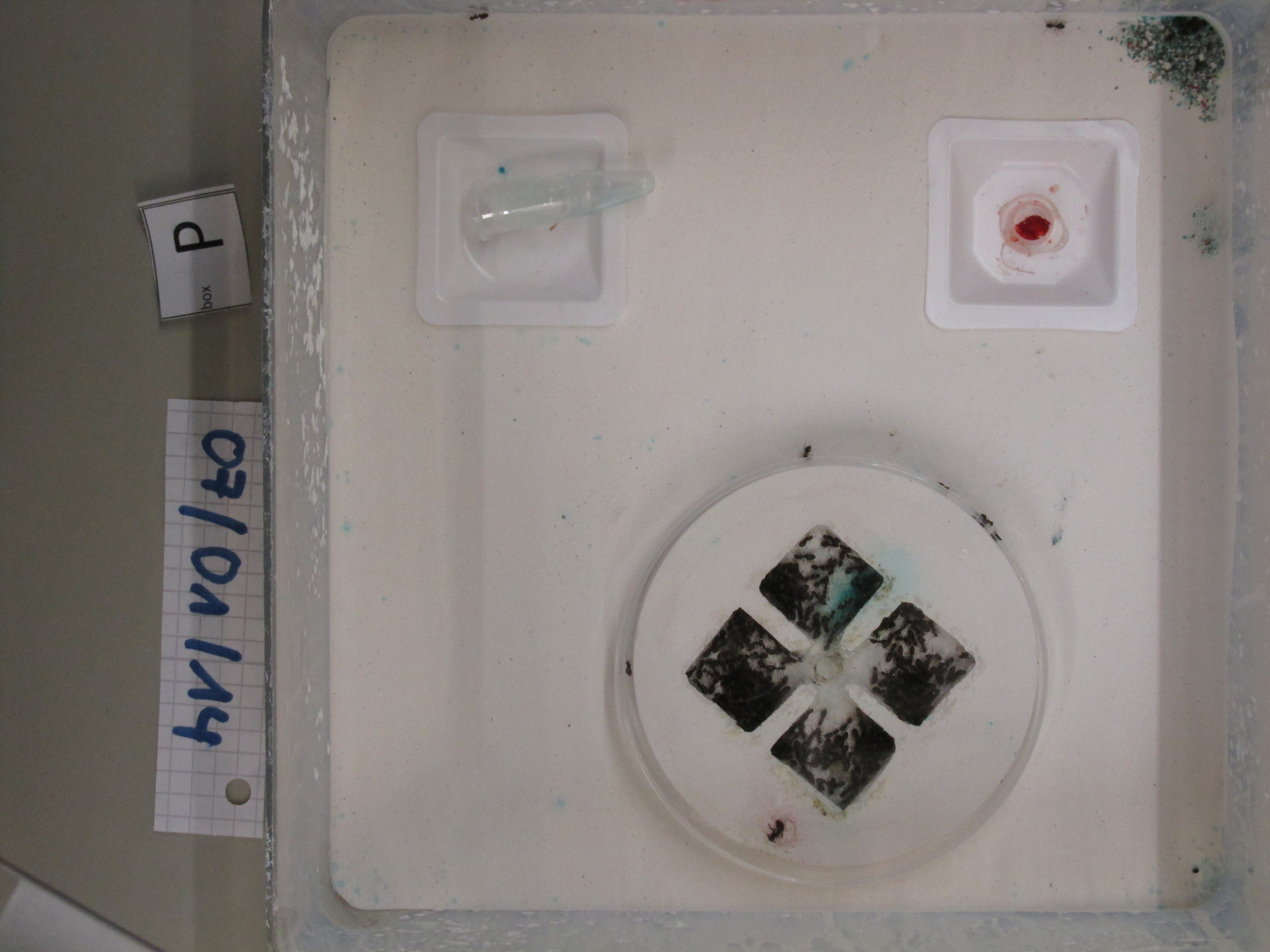

Supplement: S1 Images — Pictures of the final state of each nest and nest-box, before the ants were removed from the nest. The images were taken just before the ants were removed from the nest boxes and the photographs in Fig. 2 were taken. The dot and letter in Fig. 1 and 2 represent the “front” of the nest—i.e. the side of the nest furthest from the foraging arena wall. The opaque nest cover was removed only seconds before the photographs were taken. Similar photographs taken every week throughout the course of the experiment are available from Dryad (doi:10.5061/dryad.9fs7n). (ZIP) [file pone.0118376.s001.zip › PO 070114 (14).JPG]

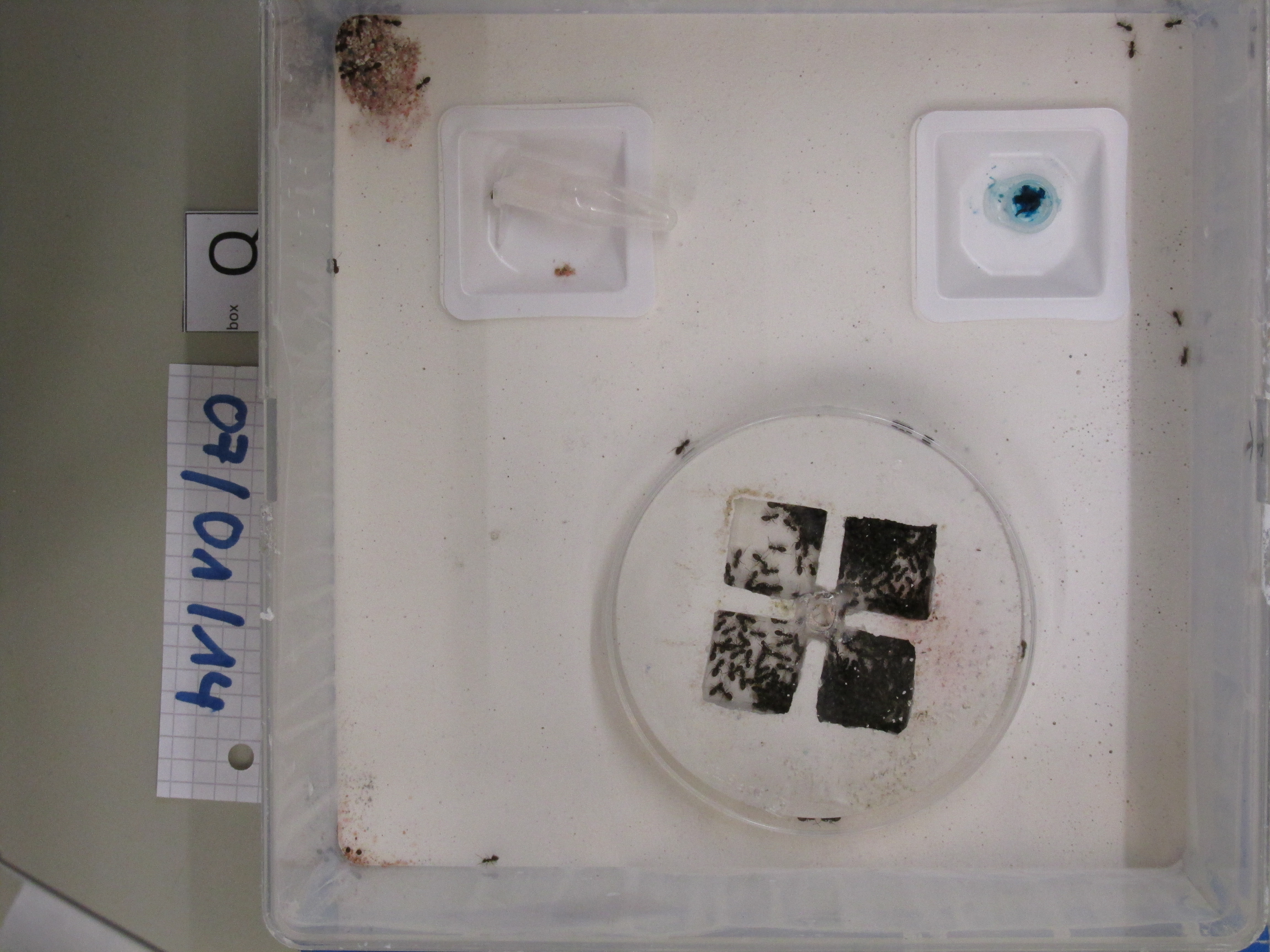

Supplement: S1 Images — Pictures of the final state of each nest and nest-box, before the ants were removed from the nest. The images were taken just before the ants were removed from the nest boxes and the photographs in Fig. 2 were taken. The dot and letter in Fig. 1 and 2 represent the “front” of the nest—i.e. the side of the nest furthest from the foraging arena wall. The opaque nest cover was removed only seconds before the photographs were taken. Similar photographs taken every week throughout the course of the experiment are available from Dryad (doi:10.5061/dryad.9fs7n). (ZIP) [file pone.0118376.s001.zip › QO 070114 (30).JPG]

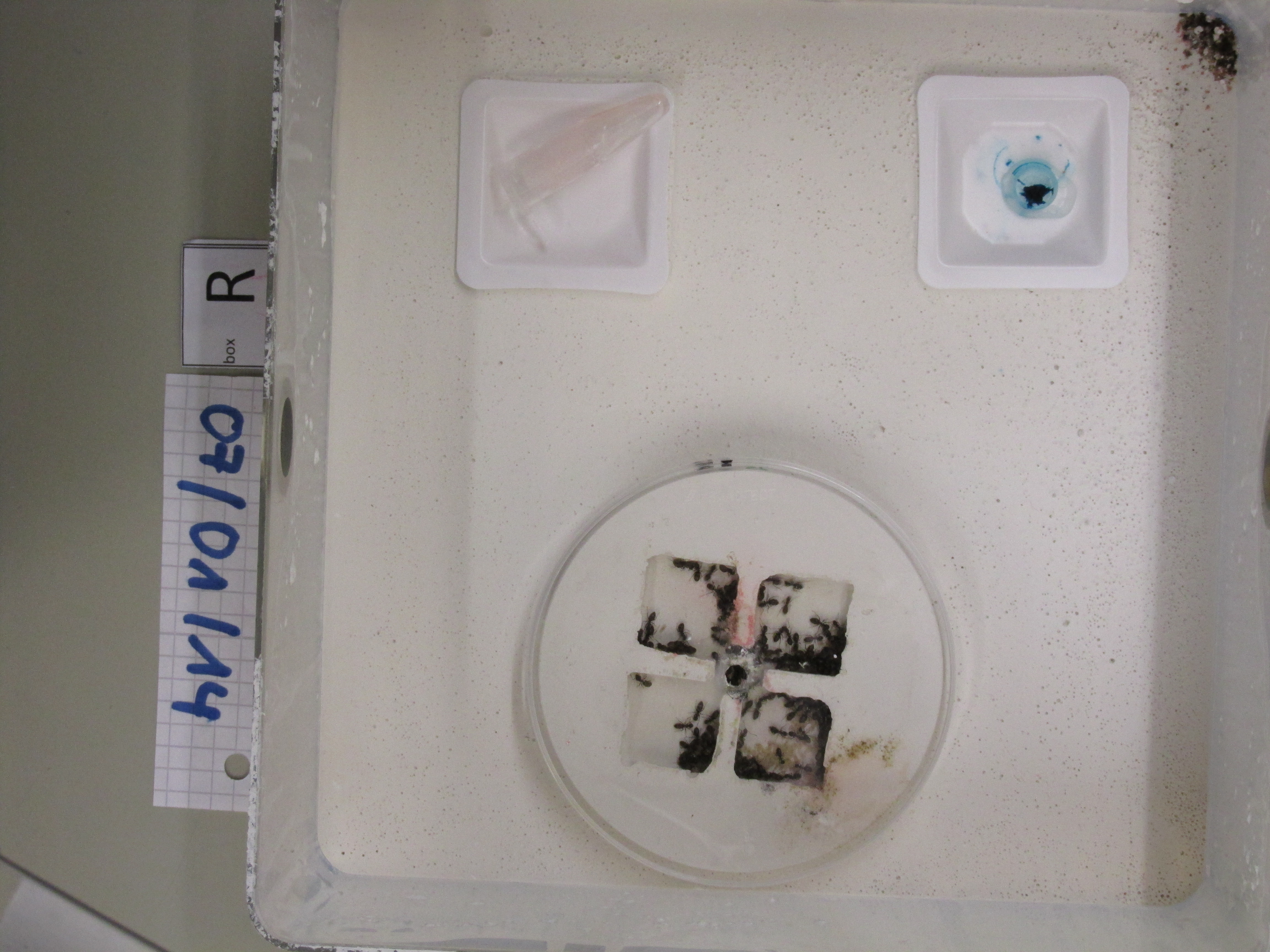

Supplement: S1 Images — Pictures of the final state of each nest and nest-box, before the ants were removed from the nest. The images were taken just before the ants were removed from the nest boxes and the photographs in Fig. 2 were taken. The dot and letter in Fig. 1 and 2 represent the “front” of the nest—i.e. the side of the nest furthest from the foraging arena wall. The opaque nest cover was removed only seconds before the photographs were taken. Similar photographs taken every week throughout the course of the experiment are available from Dryad (doi:10.5061/dryad.9fs7n). (ZIP) [file pone.0118376.s001.zip › RO 070114 (26).JPG]

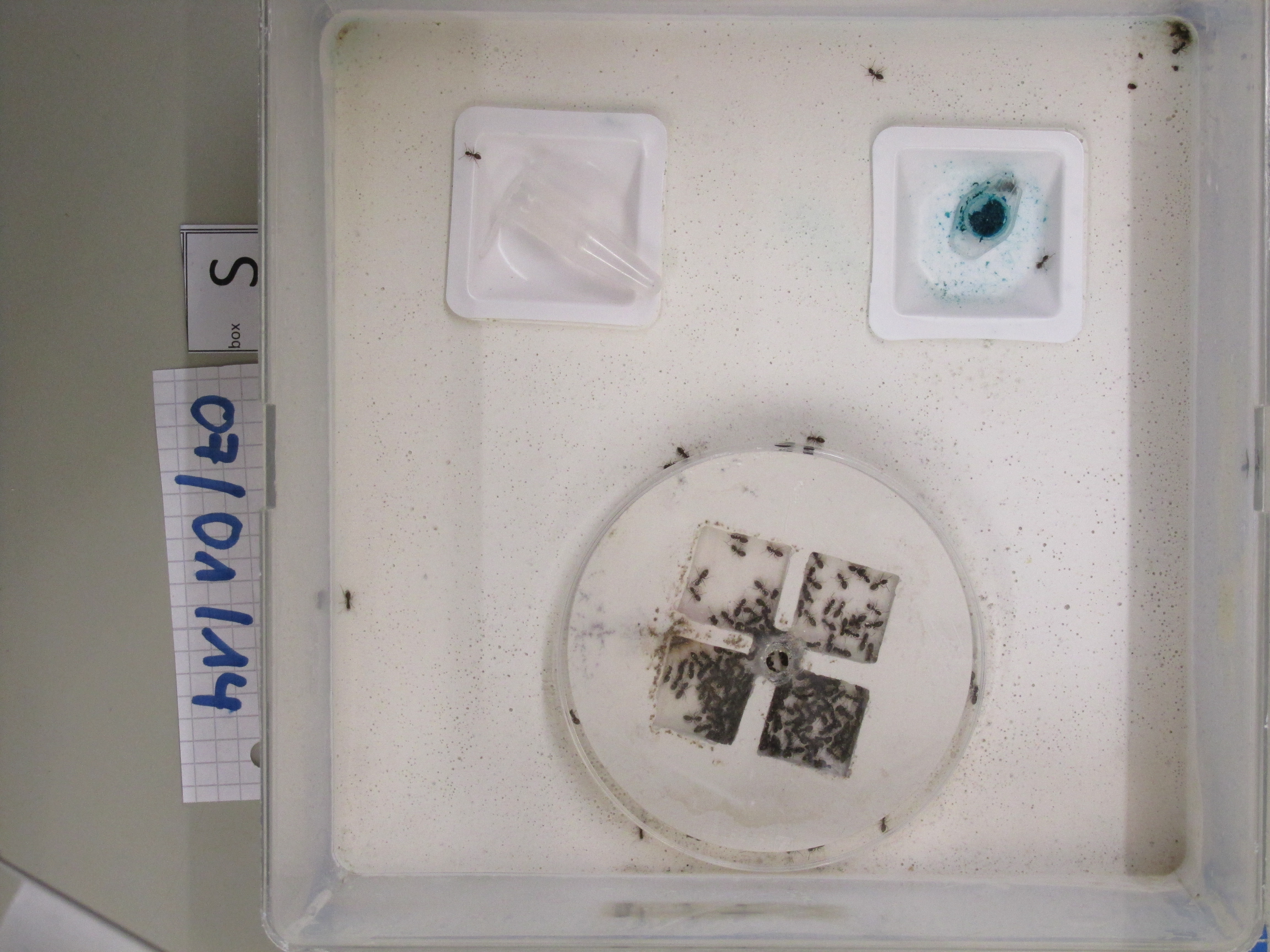

Supplement: S1 Images — Pictures of the final state of each nest and nest-box, before the ants were removed from the nest. The images were taken just before the ants were removed from the nest boxes and the photographs in Fig. 2 were taken. The dot and letter in Fig. 1 and 2 represent the “front” of the nest—i.e. the side of the nest furthest from the foraging arena wall. The opaque nest cover was removed only seconds before the photographs were taken. Similar photographs taken every week throughout the course of the experiment are available from Dryad (doi:10.5061/dryad.9fs7n). (ZIP) [file pone.0118376.s001.zip › SO 070114 (28).JPG]

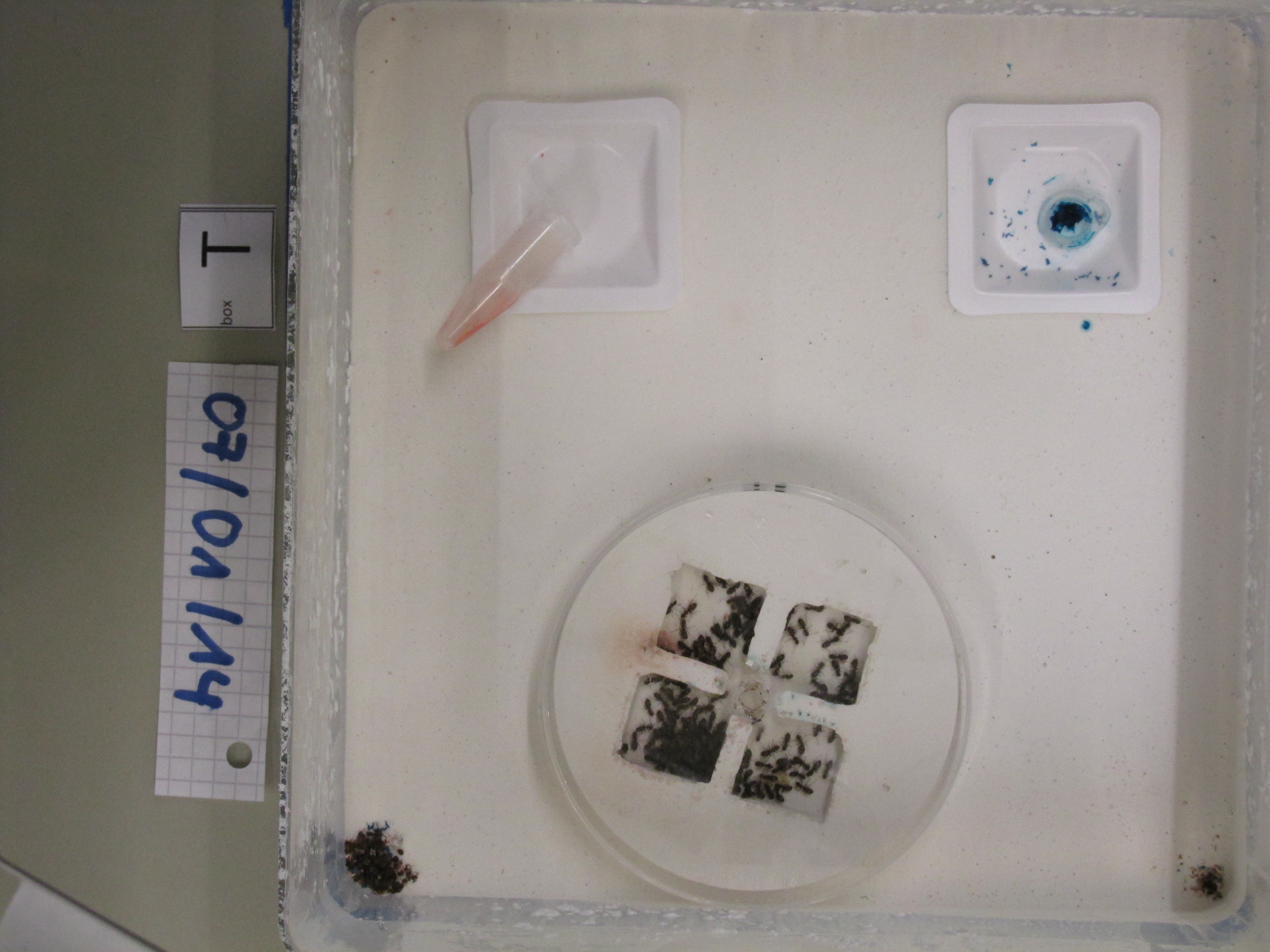

Supplement: S1 Images — Pictures of the final state of each nest and nest-box, before the ants were removed from the nest. The images were taken just before the ants were removed from the nest boxes and the photographs in Fig. 2 were taken. The dot and letter in Fig. 1 and 2 represent the “front” of the nest—i.e. the side of the nest furthest from the foraging arena wall. The opaque nest cover was removed only seconds before the photographs were taken. Similar photographs taken every week throughout the course of the experiment are available from Dryad (doi:10.5061/dryad.9fs7n). (ZIP) [file pone.0118376.s001.zip › TO 070114 (32).JPG]

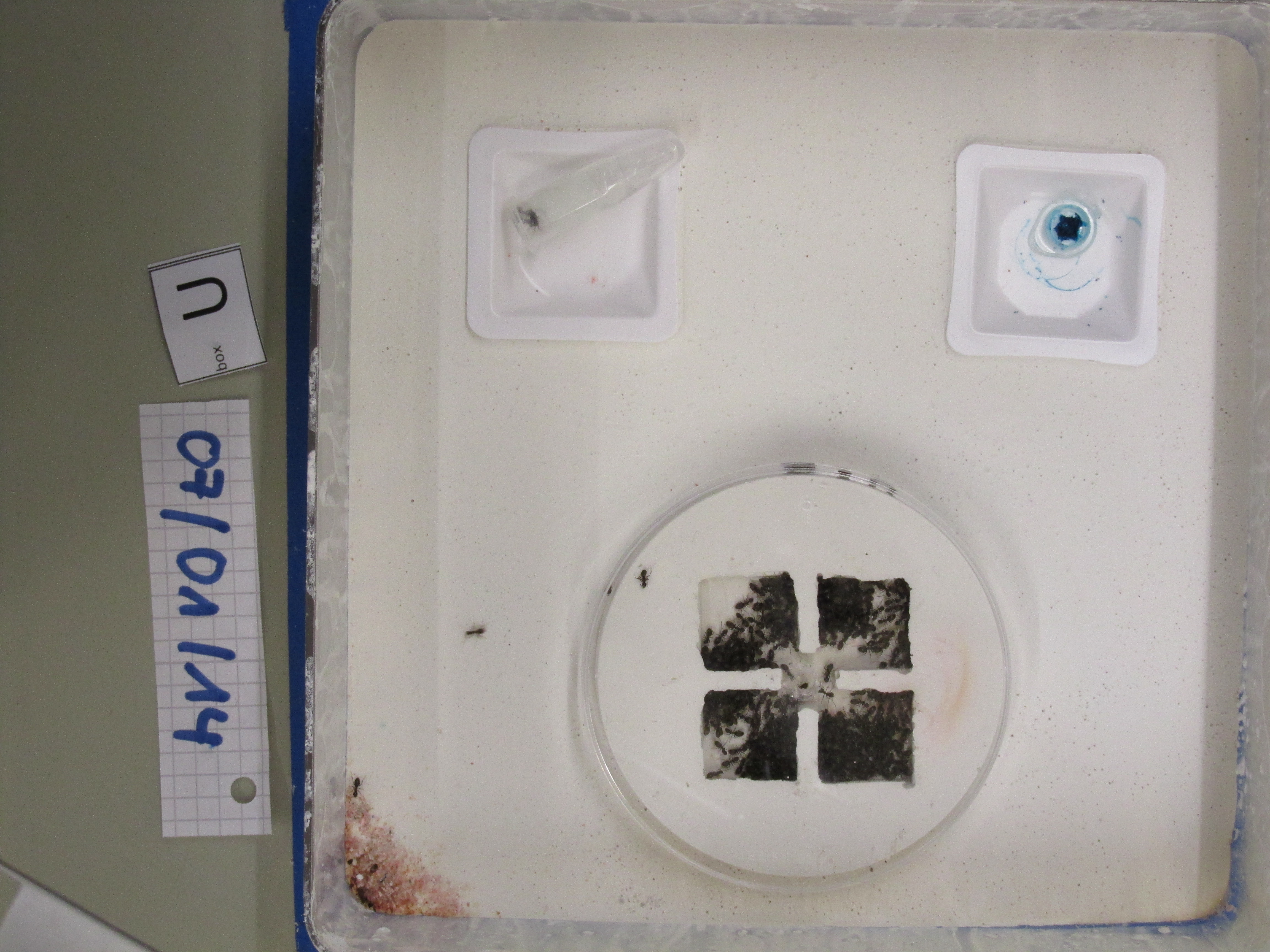

Supplement: S1 Images — Pictures of the final state of each nest and nest-box, before the ants were removed from the nest. The images were taken just before the ants were removed from the nest boxes and the photographs in Fig. 2 were taken. The dot and letter in Fig. 1 and 2 represent the “front” of the nest—i.e. the side of the nest furthest from the foraging arena wall. The opaque nest cover was removed only seconds before the photographs were taken. Similar photographs taken every week throughout the course of the experiment are available from Dryad (doi:10.5061/dryad.9fs7n). (ZIP) [file pone.0118376.s001.zip › UO 070114 (36).JPG]

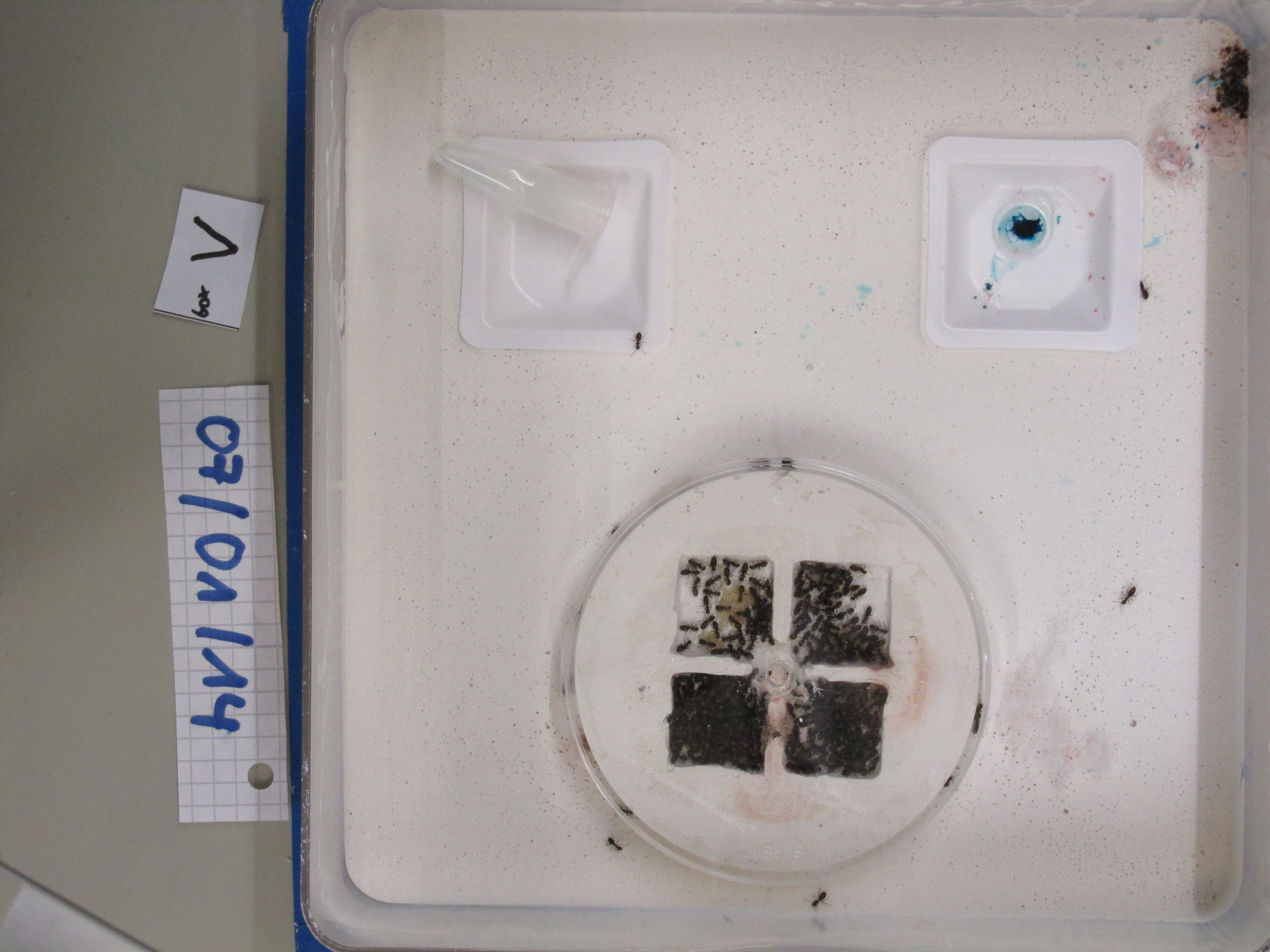

Supplement: S1 Images — Pictures of the final state of each nest and nest-box, before the ants were removed from the nest. The images were taken just before the ants were removed from the nest boxes and the photographs in Fig. 2 were taken. The dot and letter in Fig. 1 and 2 represent the “front” of the nest—i.e. the side of the nest furthest from the foraging arena wall. The opaque nest cover was removed only seconds before the photographs were taken. Similar photographs taken every week throughout the course of the experiment are available from Dryad (doi:10.5061/dryad.9fs7n). (ZIP) [file pone.0118376.s001.zip › VO 070114 (34).JPG]

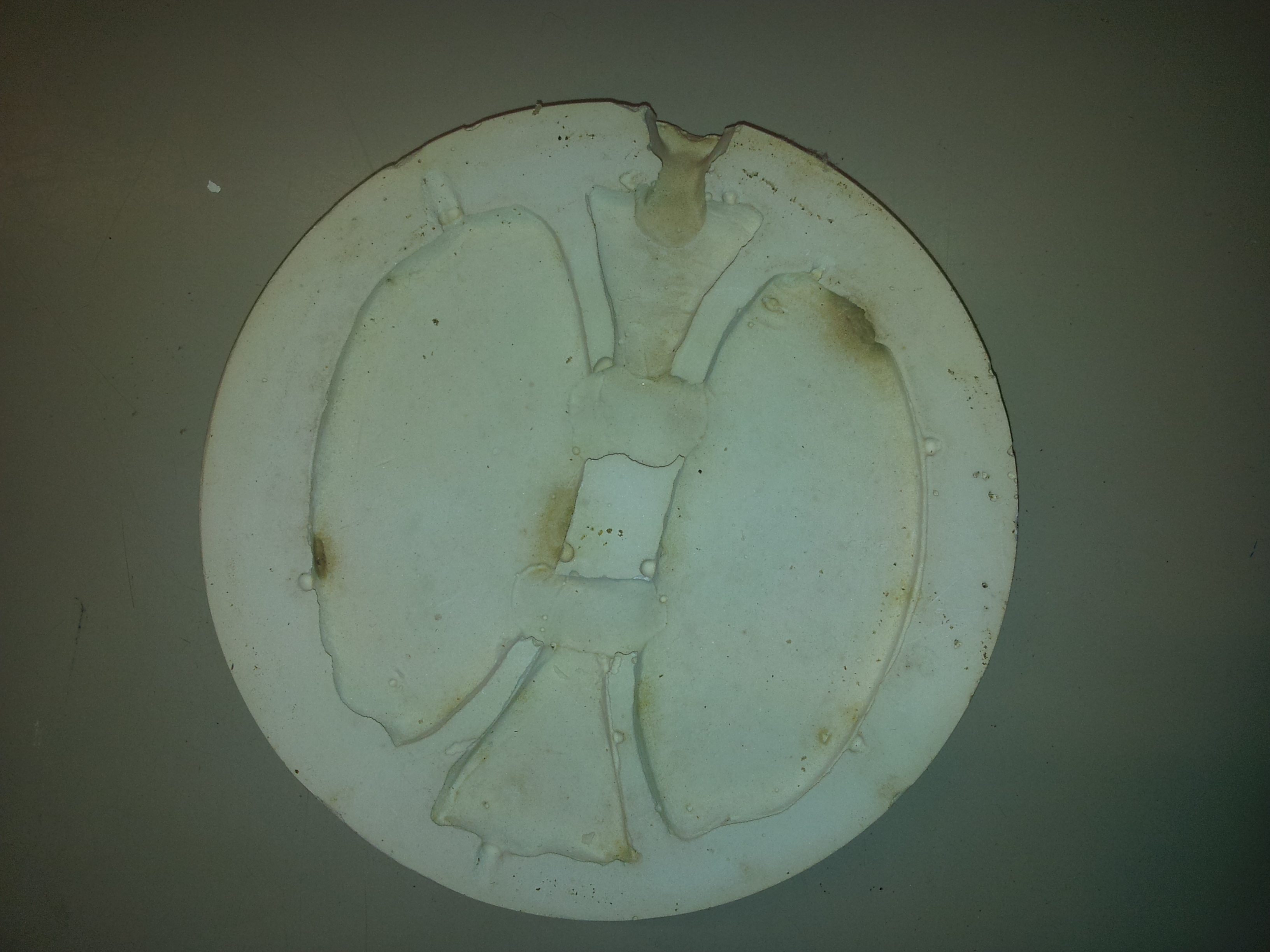

Supplement: S2 Images — We maintain larger Lasius niger colonies in our lab in plaster nests similar to those described in this study. The ants are periodically rehoused or discarded. This supplement contains a selection of images of such plaster nests, with occasional close-up pictures of the toilets. (ZIP) [file pone.0118376.s002.zip › uncoloured toilets/uncoloured ant toilet (1).jpg]

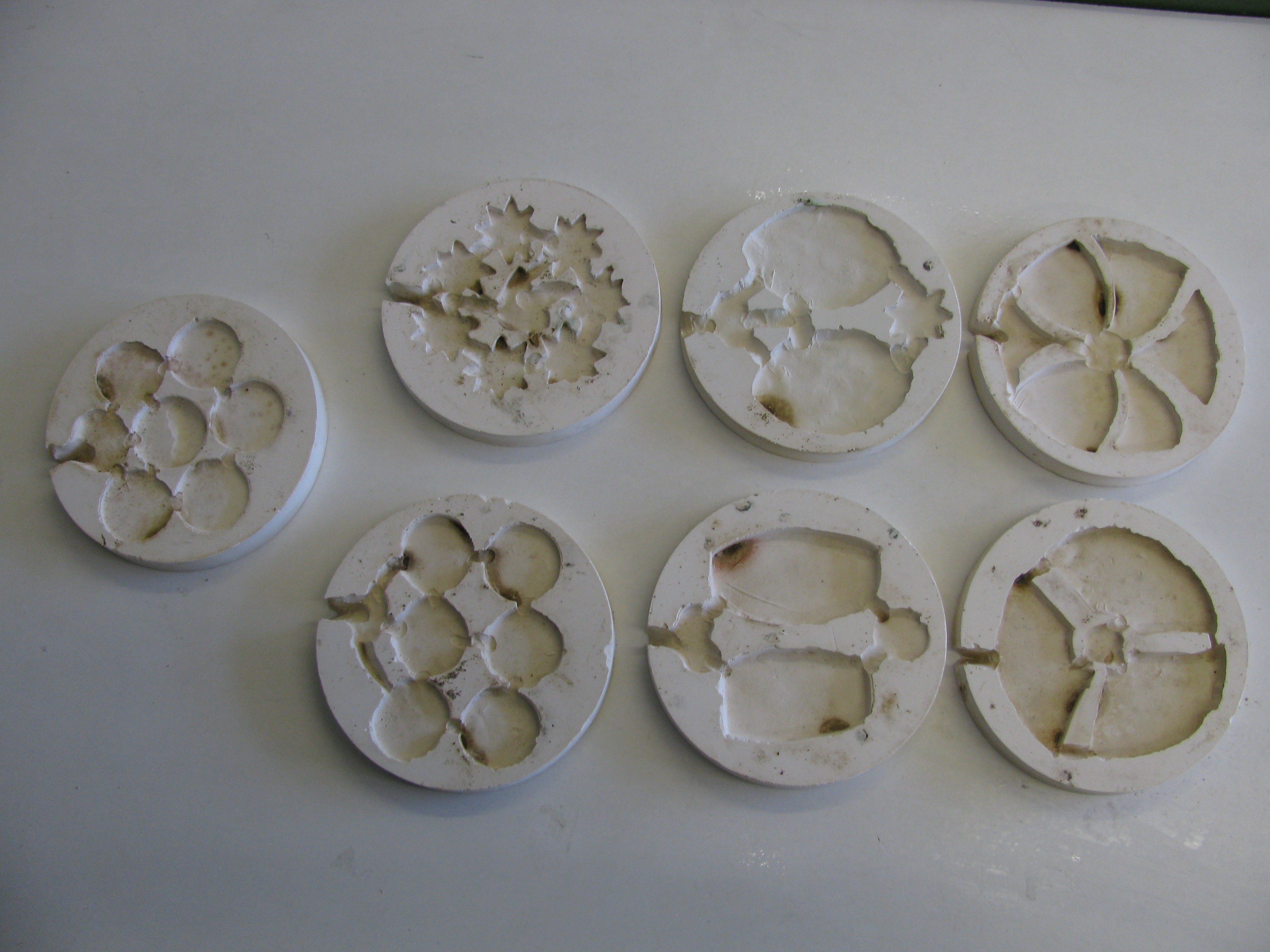

Supplement: S2 Images — We maintain larger Lasius niger colonies in our lab in plaster nests similar to those described in this study. The ants are periodically rehoused or discarded. This supplement contains a selection of images of such plaster nests, with occasional close-up pictures of the toilets. (ZIP) [file pone.0118376.s002.zip › uncoloured toilets/uncoloured ant toilet (10).JPG]

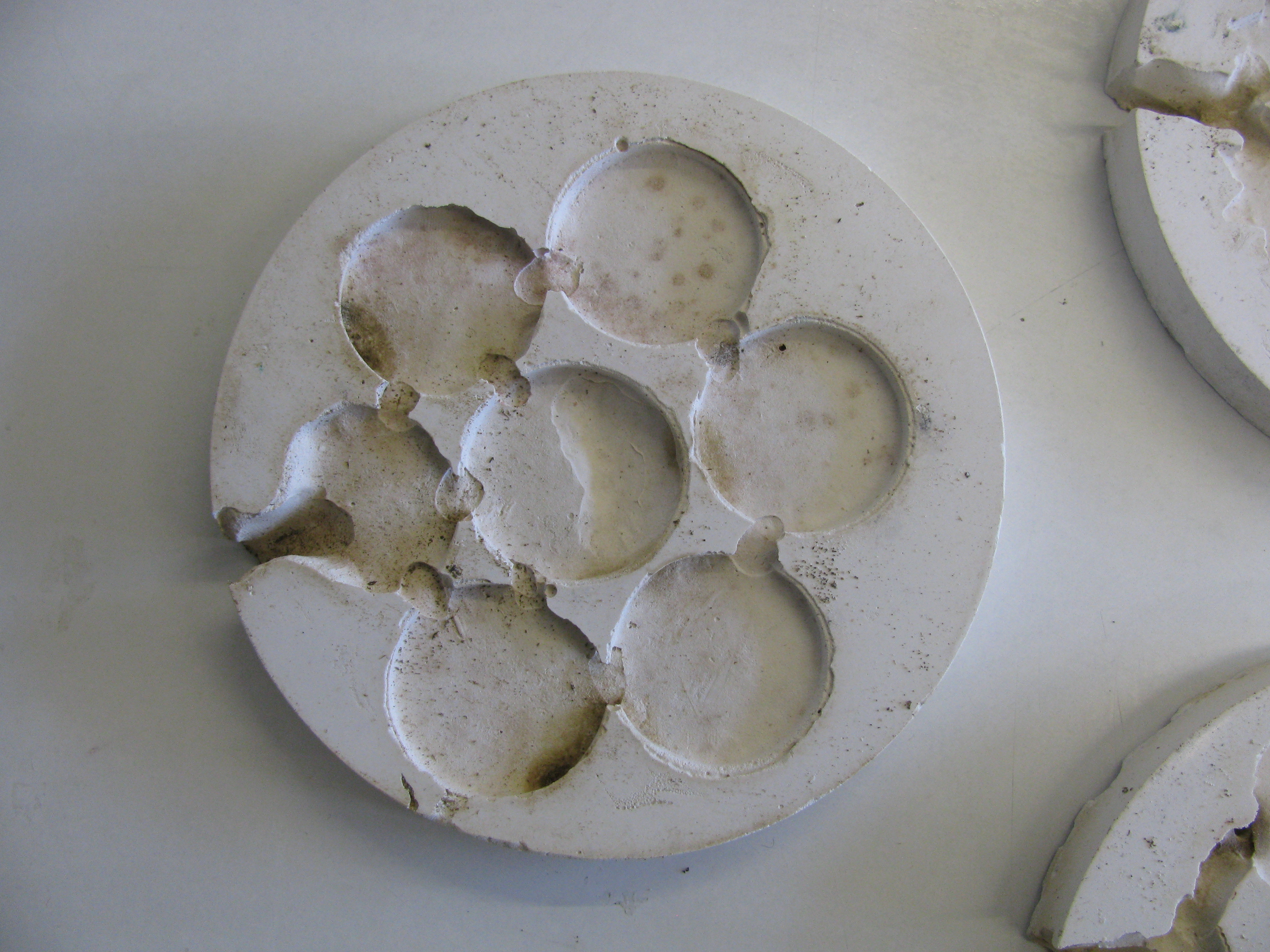

Supplement: S2 Images — We maintain larger Lasius niger colonies in our lab in plaster nests similar to those described in this study. The ants are periodically rehoused or discarded. This supplement contains a selection of images of such plaster nests, with occasional close-up pictures of the toilets. (ZIP) [file pone.0118376.s002.zip › uncoloured toilets/uncoloured ant toilet (11).JPG]

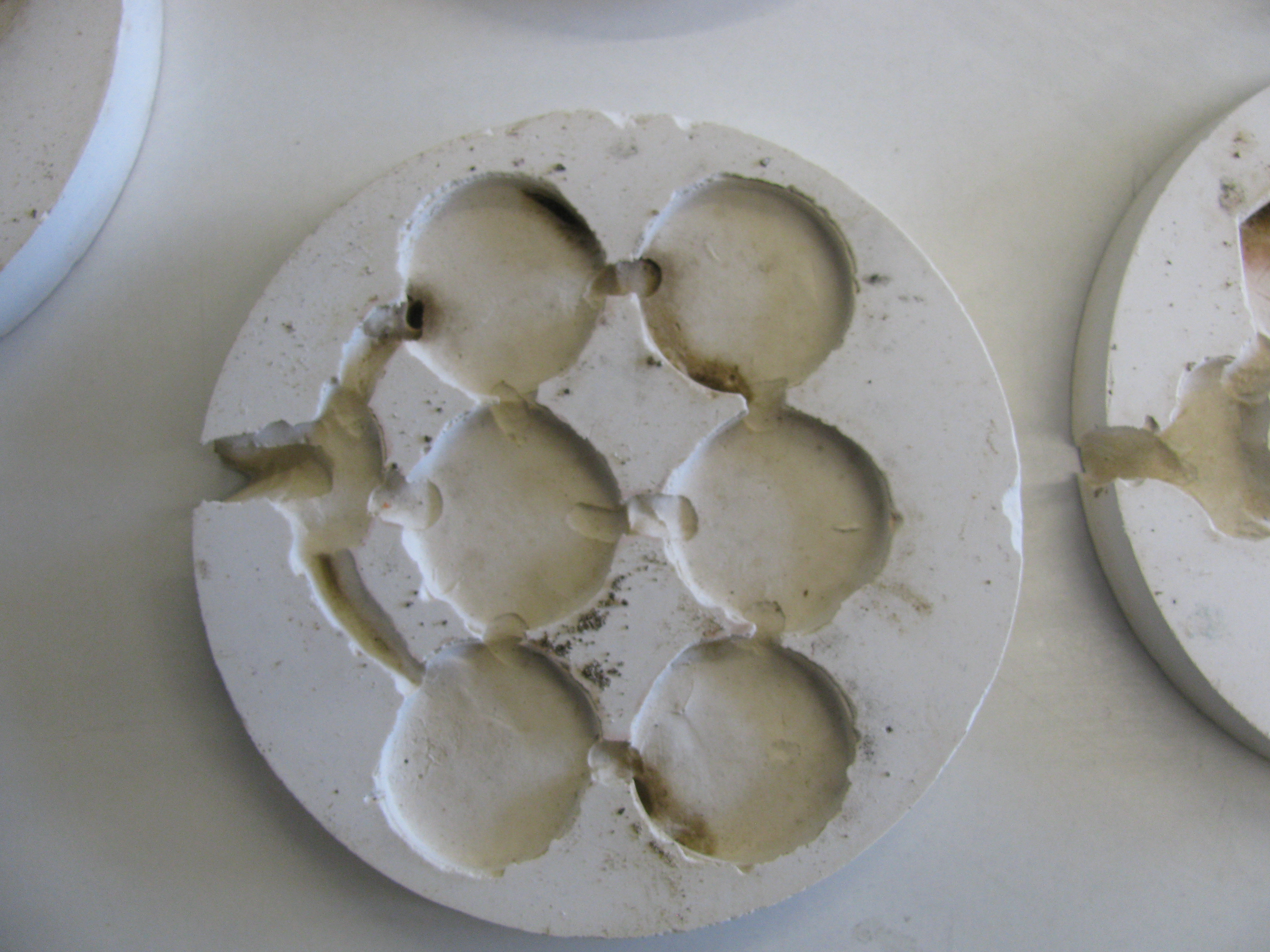

Supplement: S2 Images — We maintain larger Lasius niger colonies in our lab in plaster nests similar to those described in this study. The ants are periodically rehoused or discarded. This supplement contains a selection of images of such plaster nests, with occasional close-up pictures of the toilets. (ZIP) [file pone.0118376.s002.zip › uncoloured toilets/uncoloured ant toilet (12).JPG]

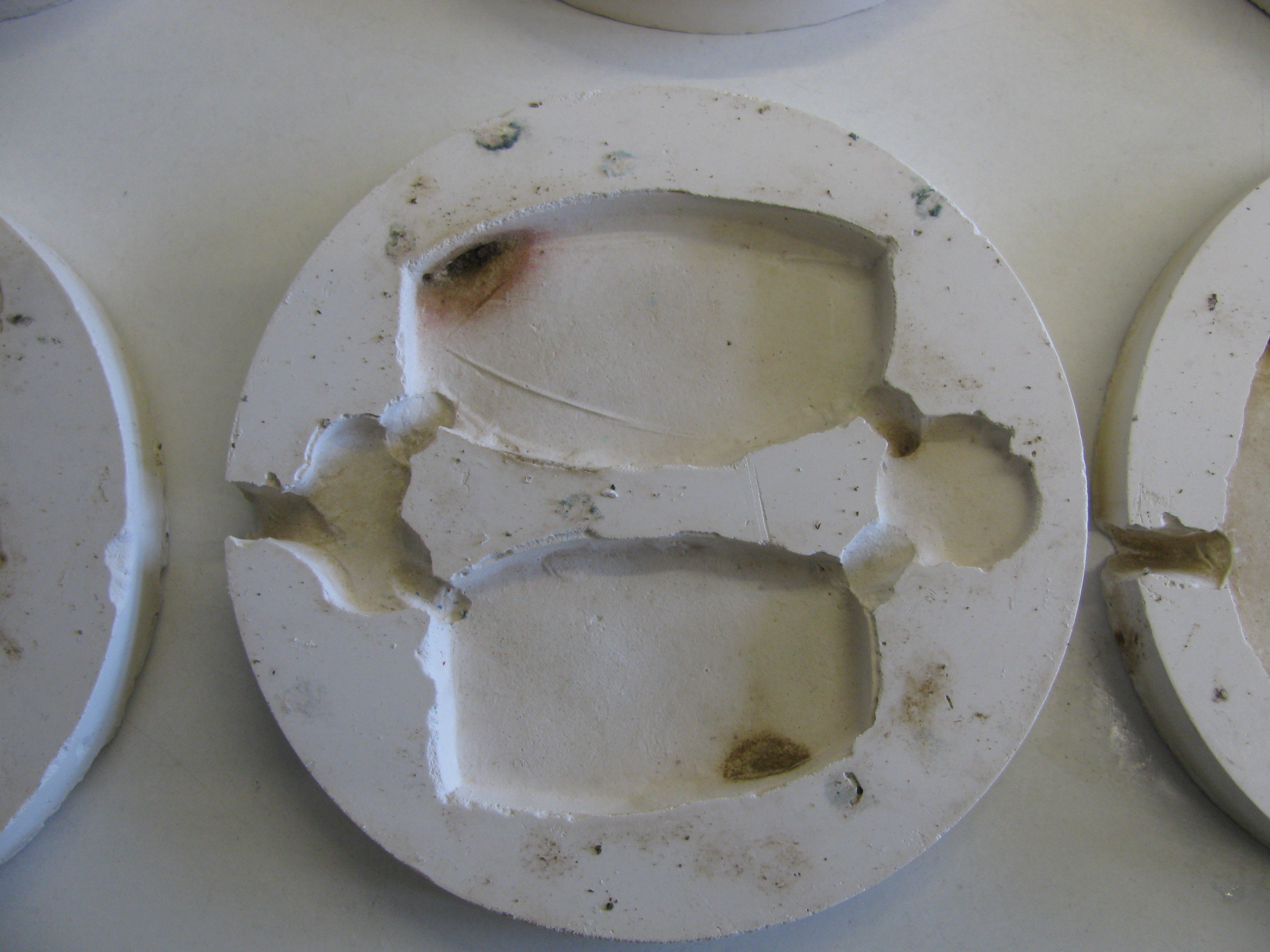

Supplement: S2 Images — We maintain larger Lasius niger colonies in our lab in plaster nests similar to those described in this study. The ants are periodically rehoused or discarded. This supplement contains a selection of images of such plaster nests, with occasional close-up pictures of the toilets. (ZIP) [file pone.0118376.s002.zip › uncoloured toilets/uncoloured ant toilet (13).JPG]

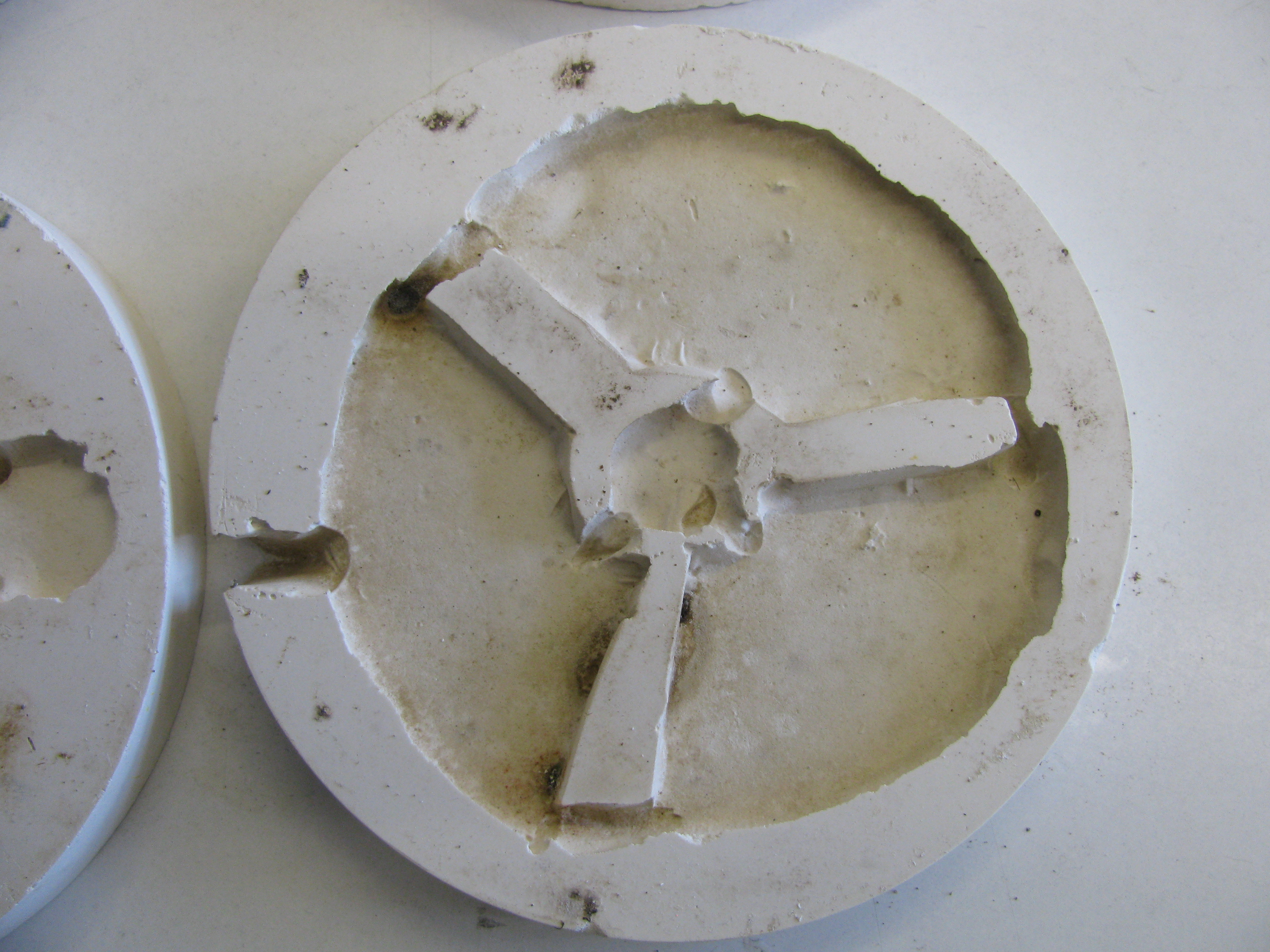

Supplement: S2 Images — We maintain larger Lasius niger colonies in our lab in plaster nests similar to those described in this study. The ants are periodically rehoused or discarded. This supplement contains a selection of images of such plaster nests, with occasional close-up pictures of the toilets. (ZIP) [file pone.0118376.s002.zip › uncoloured toilets/uncoloured ant toilet (14).JPG]

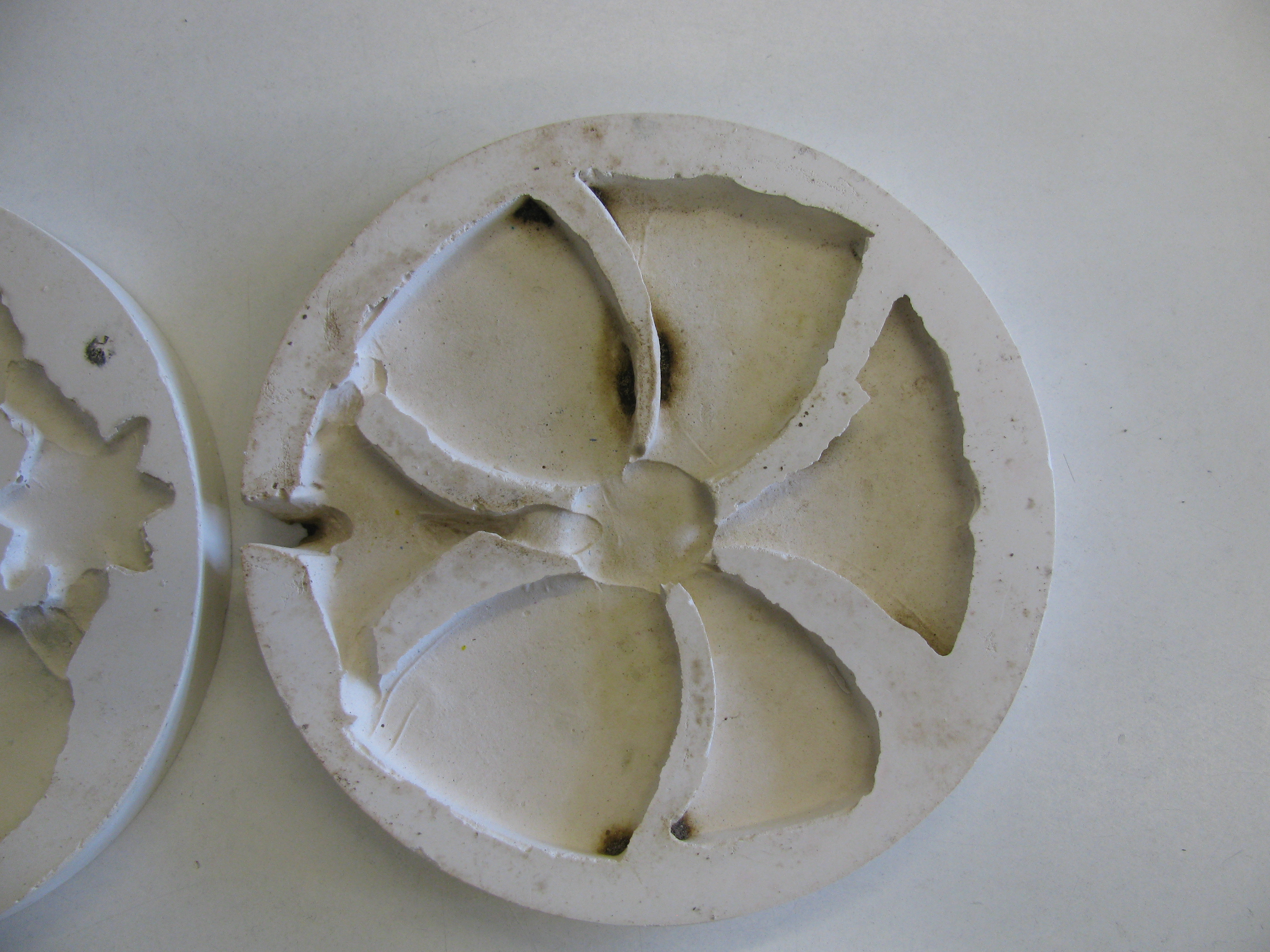

Supplement: S2 Images — We maintain larger Lasius niger colonies in our lab in plaster nests similar to those described in this study. The ants are periodically rehoused or discarded. This supplement contains a selection of images of such plaster nests, with occasional close-up pictures of the toilets. (ZIP) [file pone.0118376.s002.zip › uncoloured toilets/uncoloured ant toilet (15).JPG]

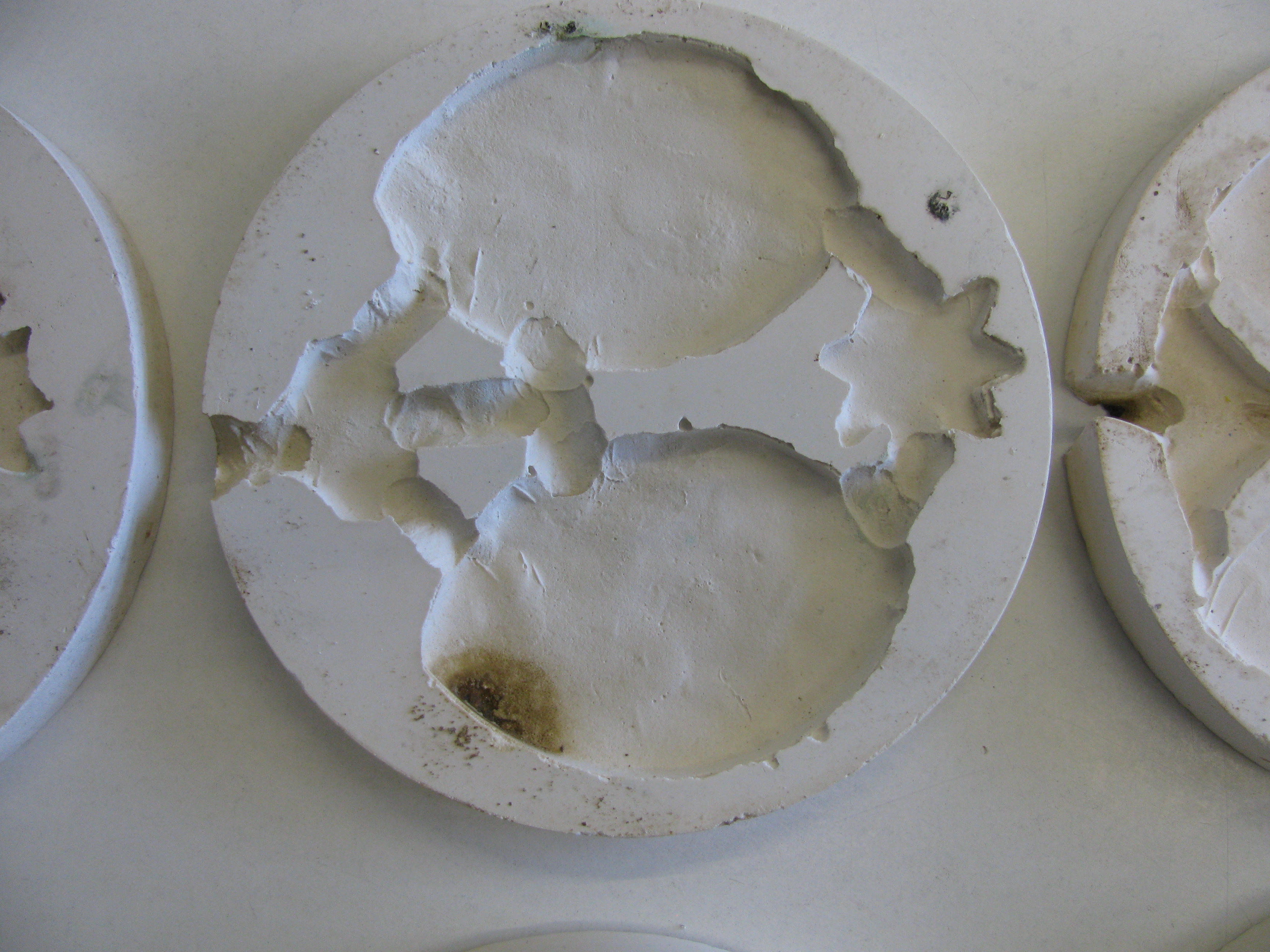

Supplement: S2 Images — We maintain larger Lasius niger colonies in our lab in plaster nests similar to those described in this study. The ants are periodically rehoused or discarded. This supplement contains a selection of images of such plaster nests, with occasional close-up pictures of the toilets. (ZIP) [file pone.0118376.s002.zip › uncoloured toilets/uncoloured ant toilet (16).JPG]

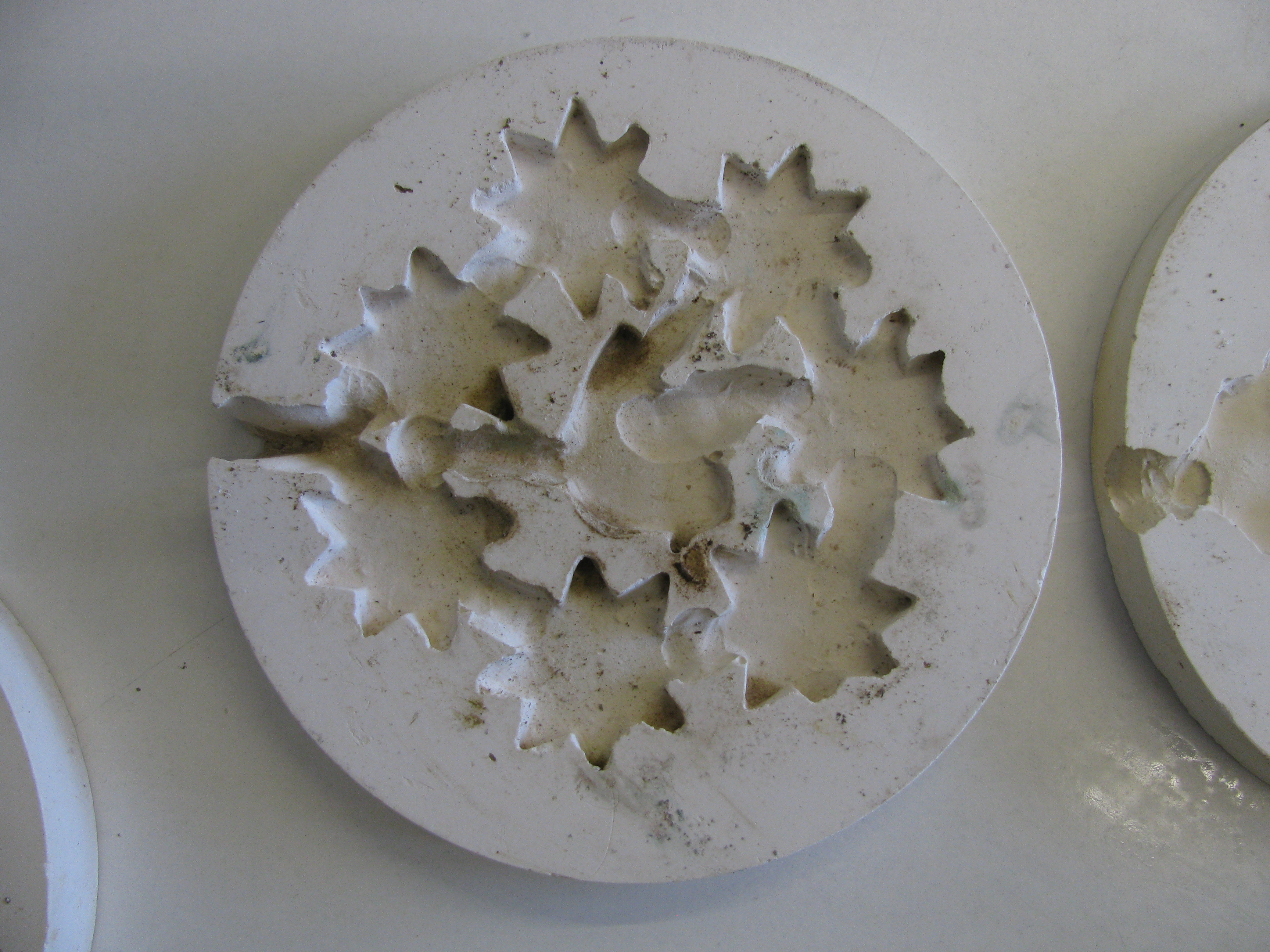

Supplement: S2 Images — We maintain larger Lasius niger colonies in our lab in plaster nests similar to those described in this study. The ants are periodically rehoused or discarded. This supplement contains a selection of images of such plaster nests, with occasional close-up pictures of the toilets. (ZIP) [file pone.0118376.s002.zip › uncoloured toilets/uncoloured ant toilet (17).JPG]

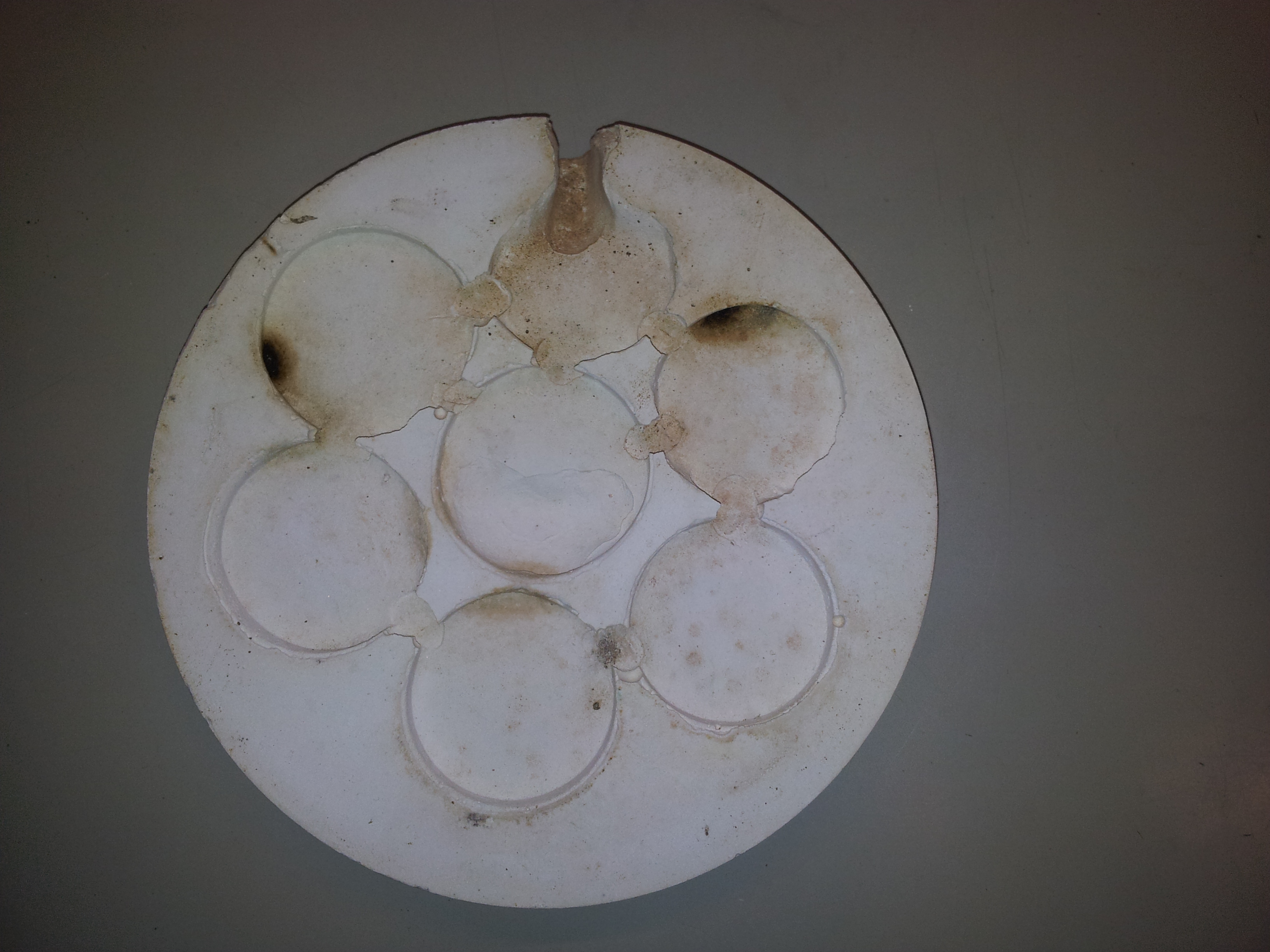

Supplement: S2 Images — We maintain larger Lasius niger colonies in our lab in plaster nests similar to those described in this study. The ants are periodically rehoused or discarded. This supplement contains a selection of images of such plaster nests, with occasional close-up pictures of the toilets. (ZIP) [file pone.0118376.s002.zip › uncoloured toilets/uncoloured ant toilet (2).jpg]

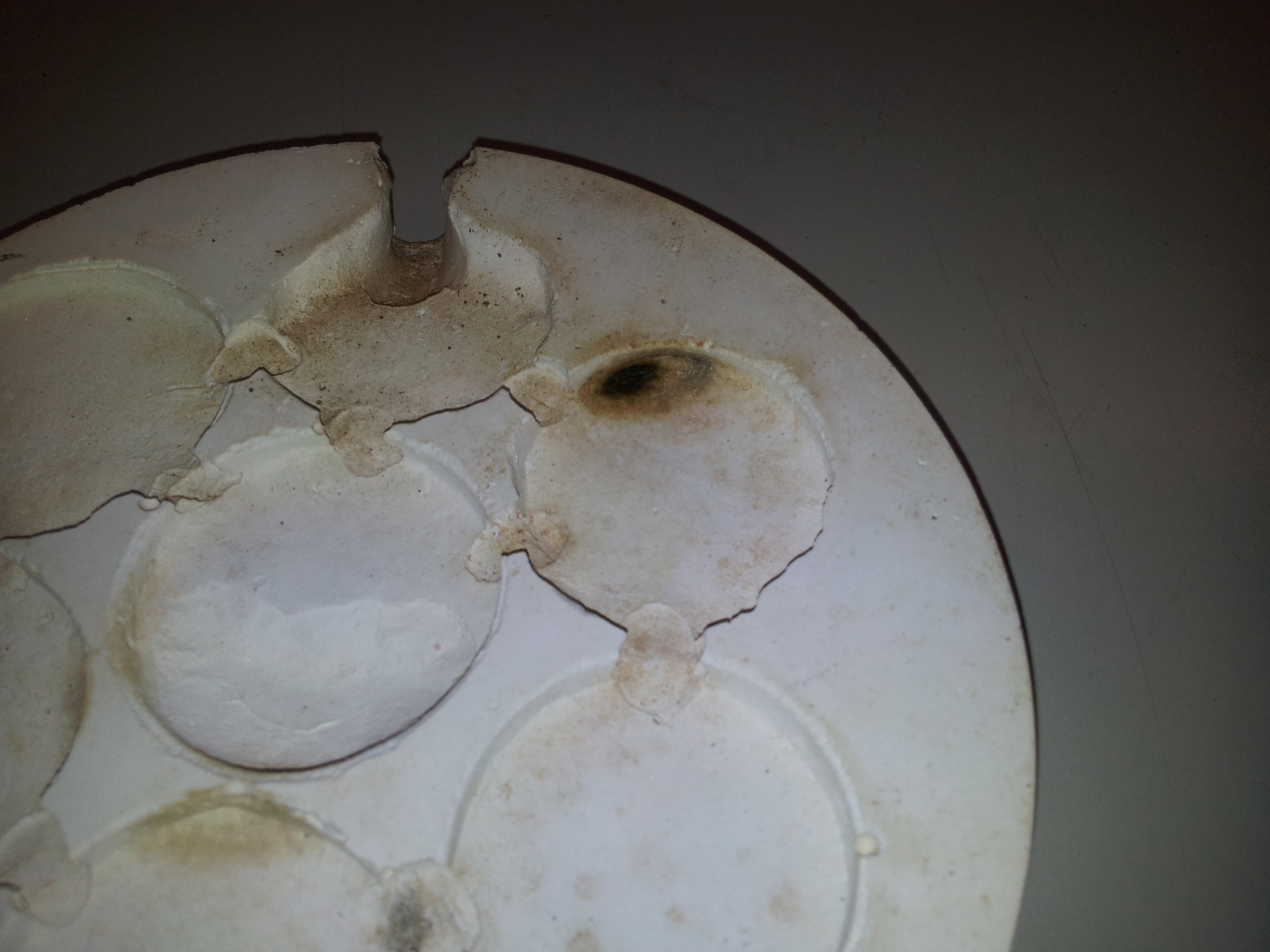

Supplement: S2 Images — We maintain larger Lasius niger colonies in our lab in plaster nests similar to those described in this study. The ants are periodically rehoused or discarded. This supplement contains a selection of images of such plaster nests, with occasional close-up pictures of the toilets. (ZIP) [file pone.0118376.s002.zip › uncoloured toilets/uncoloured ant toilet (3).jpg]

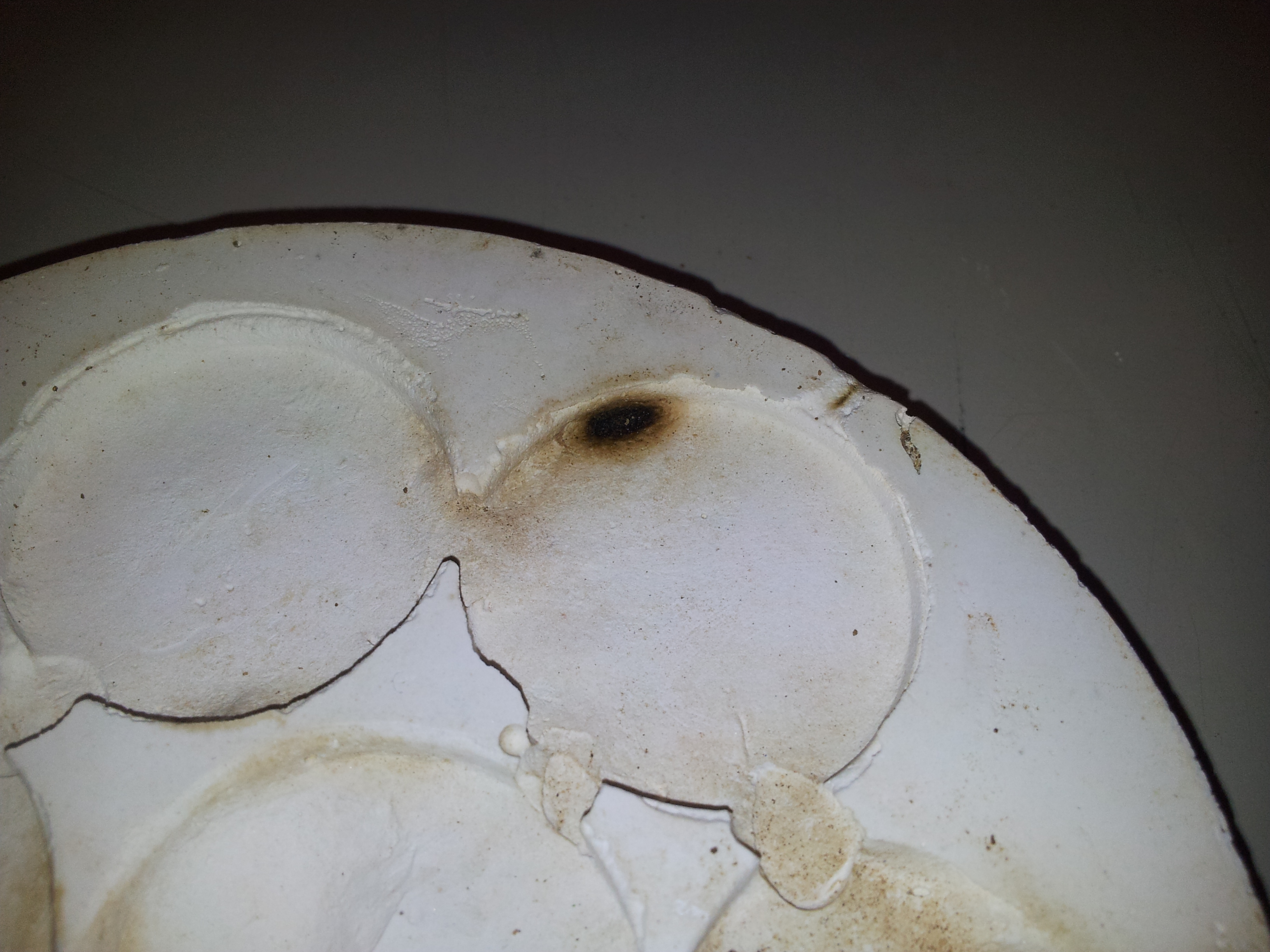

Supplement: S2 Images — We maintain larger Lasius niger colonies in our lab in plaster nests similar to those described in this study. The ants are periodically rehoused or discarded. This supplement contains a selection of images of such plaster nests, with occasional close-up pictures of the toilets. (ZIP) [file pone.0118376.s002.zip › uncoloured toilets/uncoloured ant toilet (4).jpg]

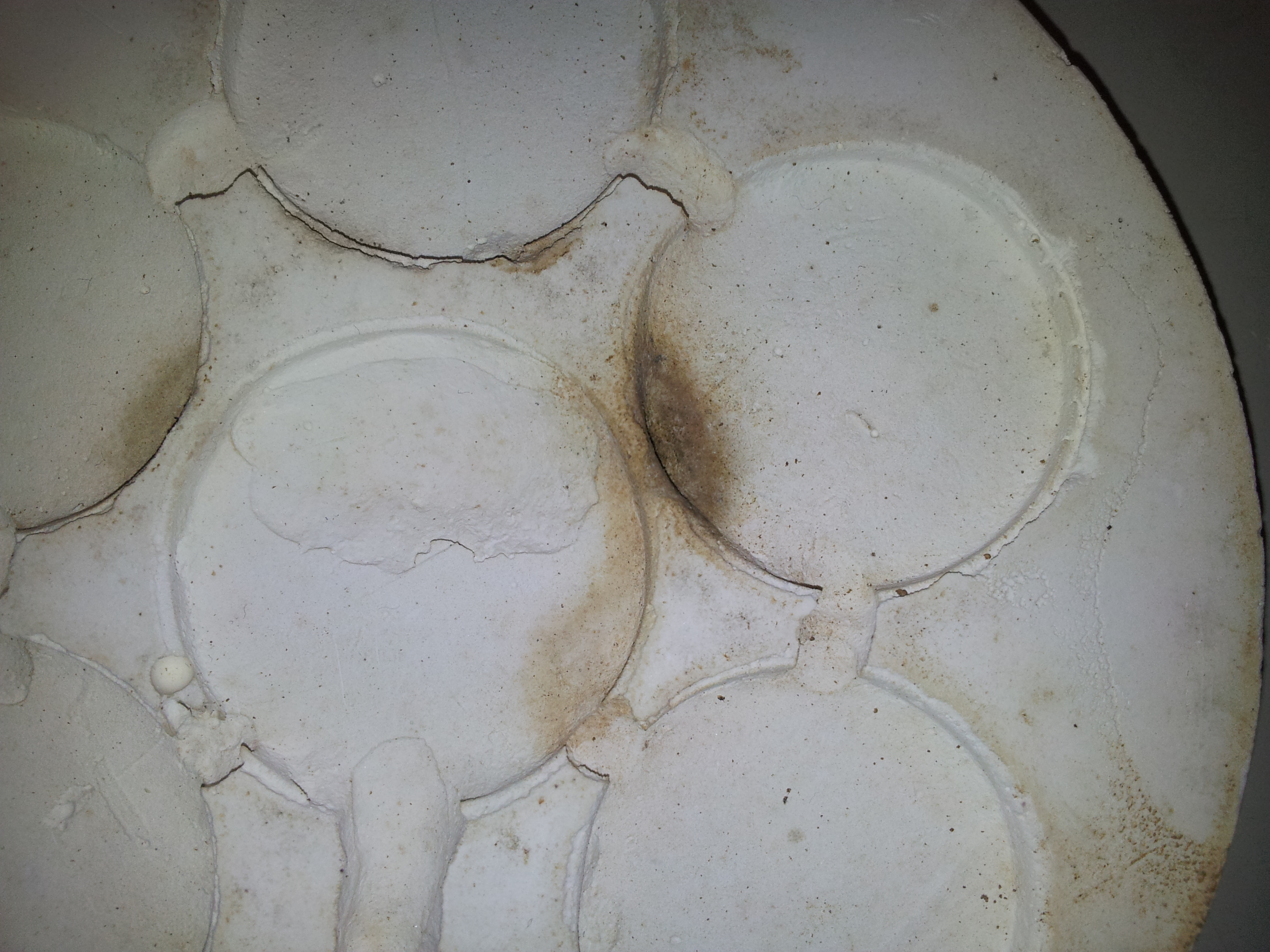

Supplement: S2 Images — We maintain larger Lasius niger colonies in our lab in plaster nests similar to those described in this study. The ants are periodically rehoused or discarded. This supplement contains a selection of images of such plaster nests, with occasional close-up pictures of the toilets. (ZIP) [file pone.0118376.s002.zip › uncoloured toilets/uncoloured ant toilet (5).jpg]

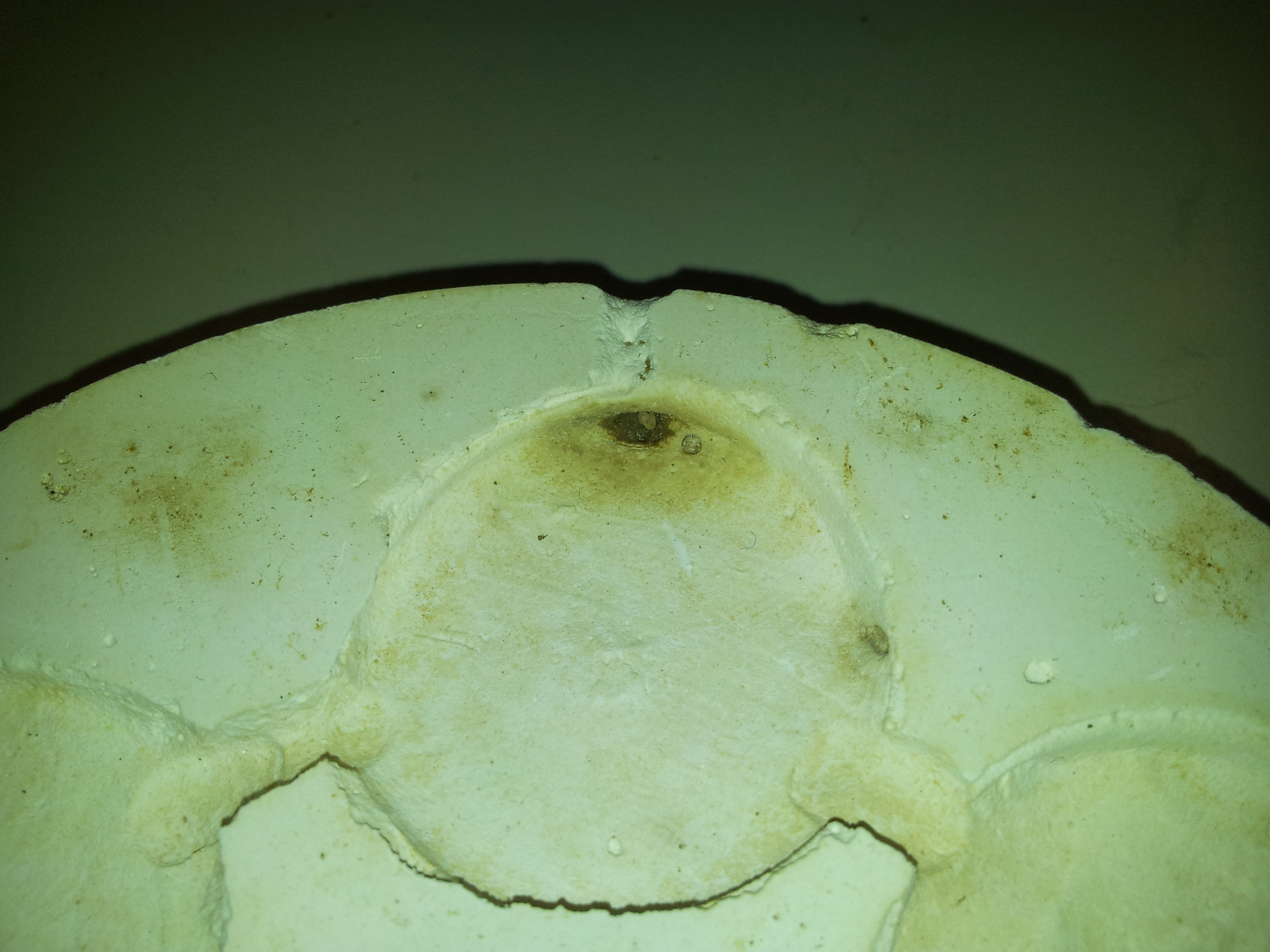

Supplement: S2 Images — We maintain larger Lasius niger colonies in our lab in plaster nests similar to those described in this study. The ants are periodically rehoused or discarded. This supplement contains a selection of images of such plaster nests, with occasional close-up pictures of the toilets. (ZIP) [file pone.0118376.s002.zip › uncoloured toilets/uncoloured ant toilet (6).jpg]

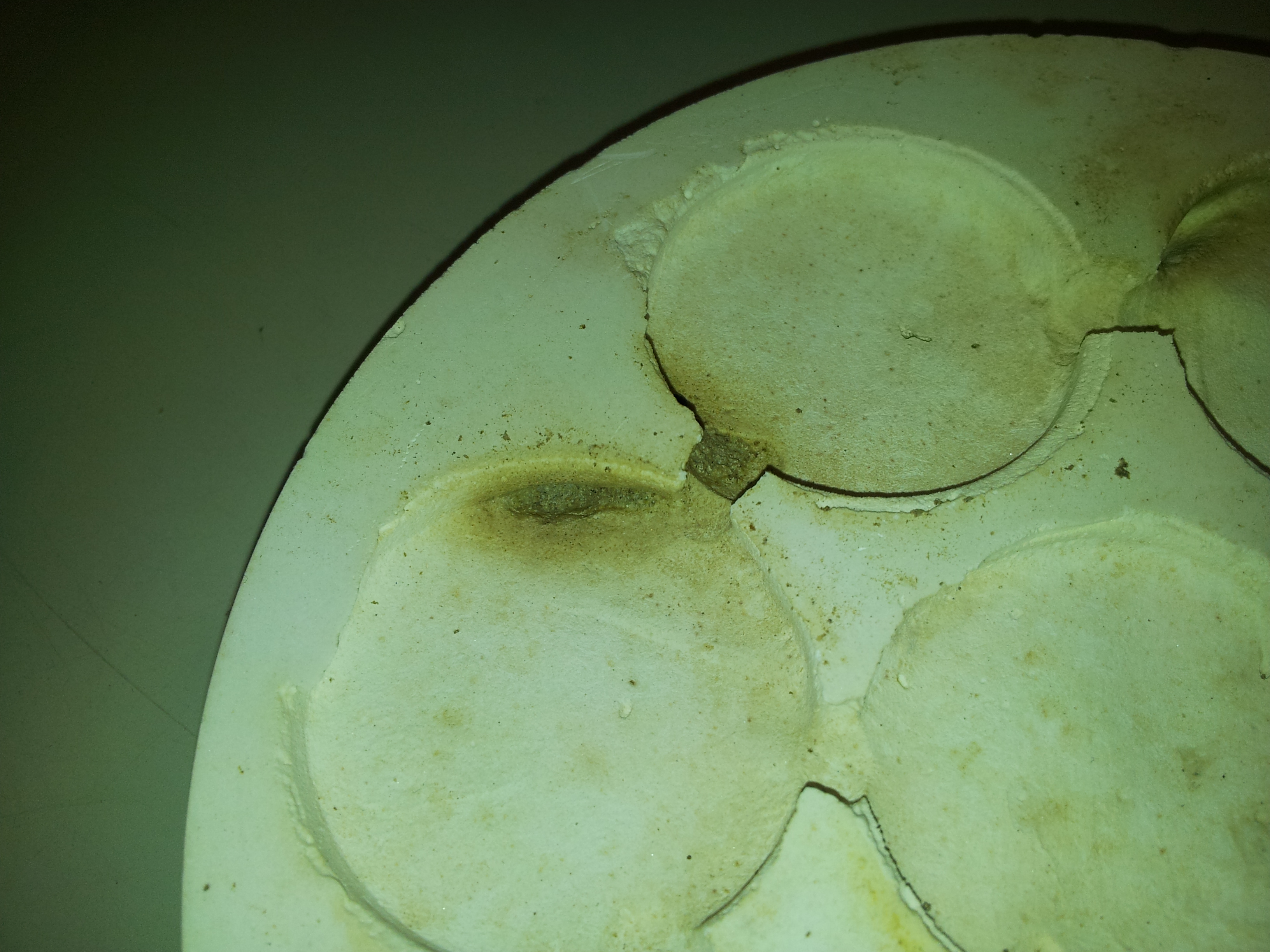

Supplement: S2 Images — We maintain larger Lasius niger colonies in our lab in plaster nests similar to those described in this study. The ants are periodically rehoused or discarded. This supplement contains a selection of images of such plaster nests, with occasional close-up pictures of the toilets. (ZIP) [file pone.0118376.s002.zip › uncoloured toilets/uncoloured ant toilet (7).jpg]

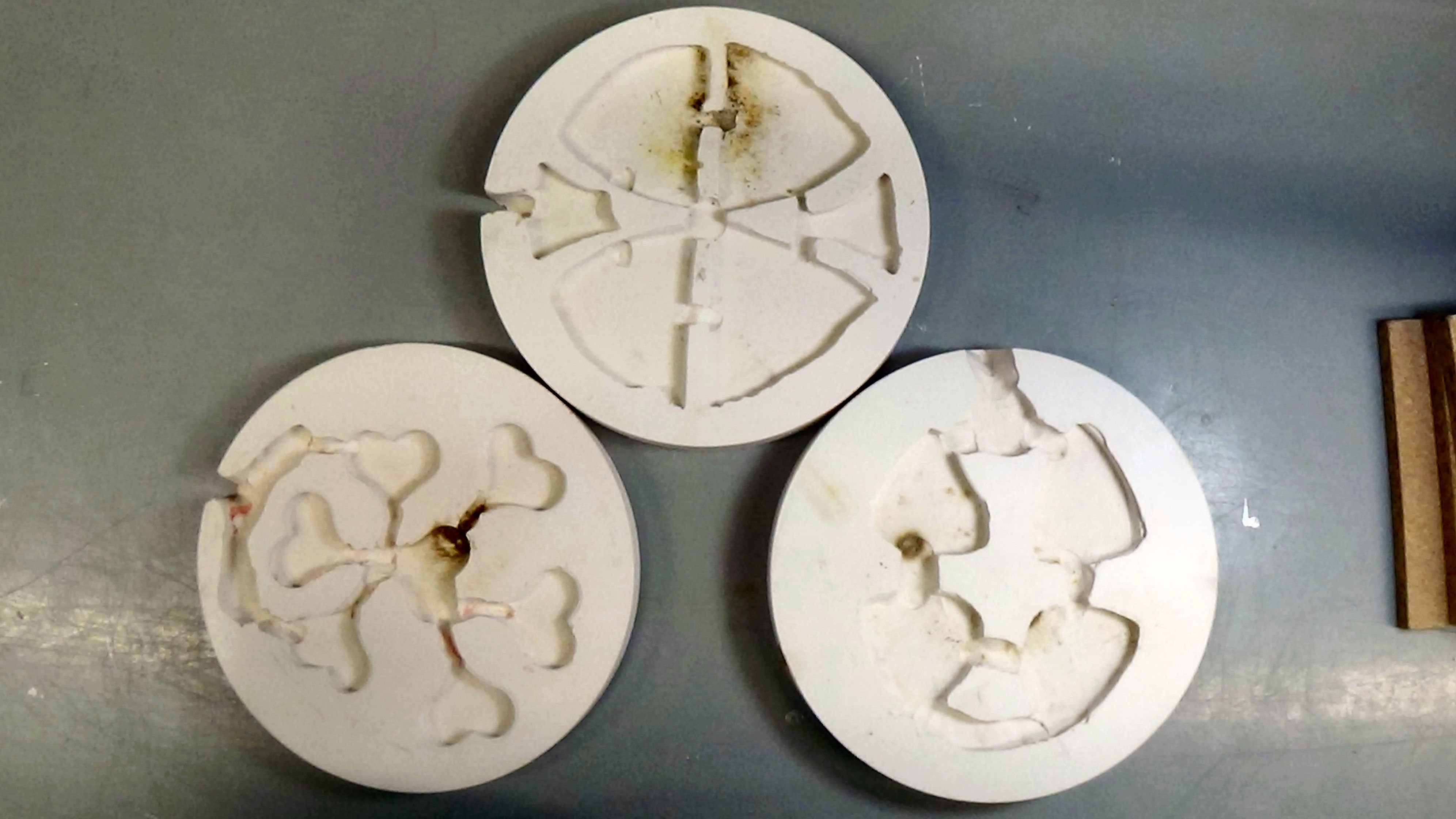

Supplement: S2 Images — We maintain larger Lasius niger colonies in our lab in plaster nests similar to those described in this study. The ants are periodically rehoused or discarded. This supplement contains a selection of images of such plaster nests, with occasional close-up pictures of the toilets. (ZIP) [file pone.0118376.s002.zip › uncoloured toilets/uncoloured ant toilet (8).JPG]

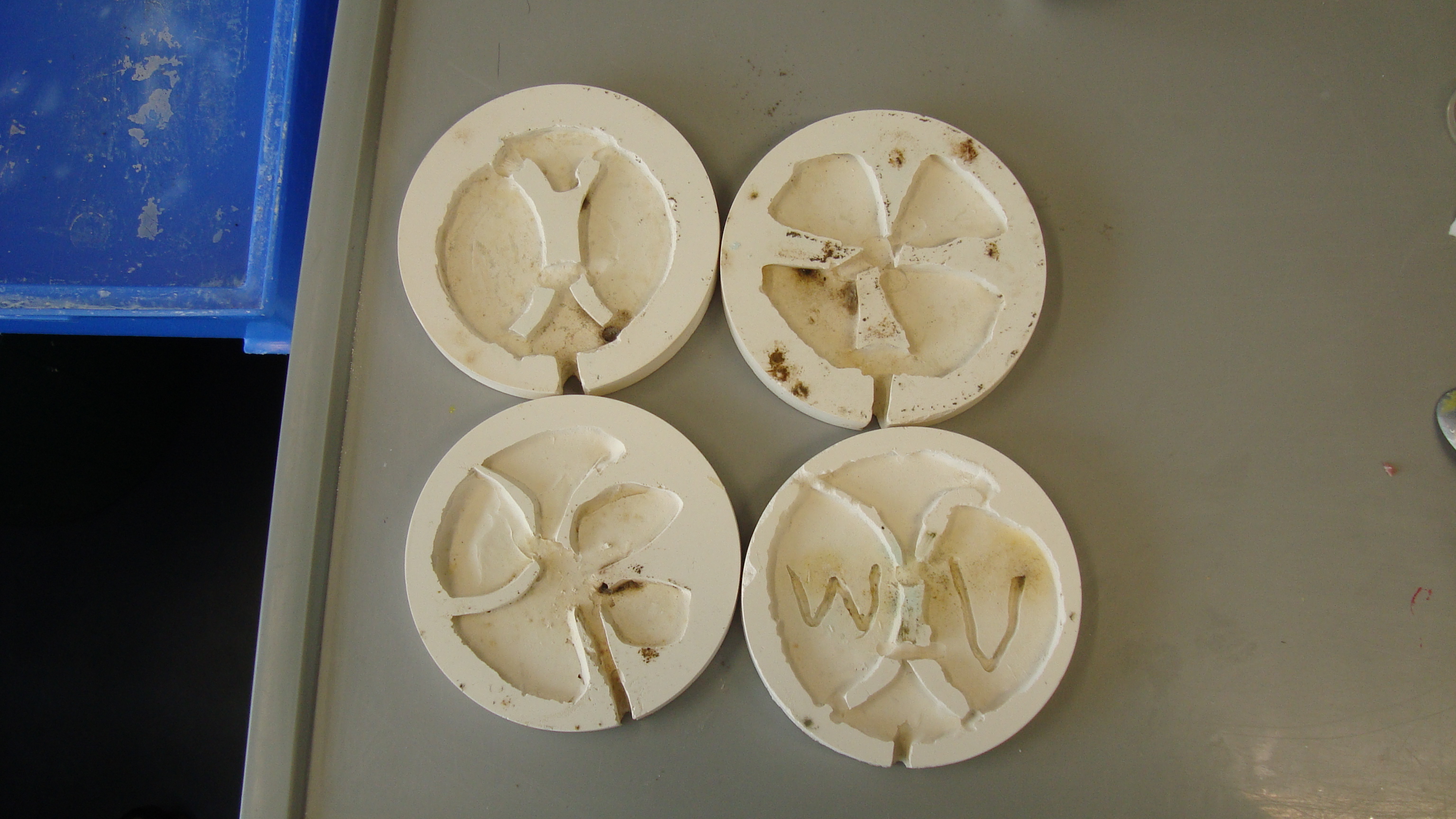

Supplement: S2 Images — We maintain larger Lasius niger colonies in our lab in plaster nests similar to those described in this study. The ants are periodically rehoused or discarded. This supplement contains a selection of images of such plaster nests, with occasional close-up pictures of the toilets. (ZIP) [file pone.0118376.s002.zip › uncoloured toilets/uncoloured ant toilet (9).JPG]
