# Supplementary material for: Nest Etiquette—Where Ants Go When Nature Calls
Source: PLoS One. 2015 Feb 18;10(2):e0118376. doi: 10.1371/journal.pone.0118376 (PMC4332866; doi:10.1371/journal.pone.0118376)
Supplement: S1 Methods — Detailed methods including details of ant maintenance, food and nest preparation, data collections, and the instructions given to the blind observer. (PDF) [file pone.0118376.s003.pdf]

The following electronic supplementary material comprises a detailed description of the methods for The paper : Nest etiquette - where ants go when nature calls, by Tomer J. Czaczkes*, Jürgen Heinze and Joachim Ruther

*corresponding author, email tomer.czaczkes@ur.de

Methods

Nest design and ant maintenance

21 groups of 150-300 ants, along with small amounts of brood (henceforth colonies) were removed from their mother colonies (13 mother colonies, 1-2 colonies per mother colony) and housed in circular plaster nests 9 cm in diameter. Each plaster nest contained a cavity c.3 mm high in which the ants lived. The cavity consisted of four square chambers 20 x 20 mm, connected via one corner to an entrance chamber 10 x 10 mm (see Fig. 1 in main text). The nest was covered by a clear plastic lid. A second plastic lid, which was made opaque using a thin layer of plaster, was placed over the first lid. A hole in the centre of the lids served as an entrance to the nest, via the entrance chamber. This nest design ensured that all chambers were equidistant from the entrance, and also removed the need for tunnels between chambers. White plaster was used at it provides a good contrast with coloured or dark material, and regulates humidity levels in the nest, absorbing excess moisture and releasing it if the air becomes too dry.

Each colony was housed in a foraging box (19x19 cm), whose sides were coated in fluon (Whitford GmbH) to prevent the ants from escaping. Each colony was provided with a carbohydrate source and a protein source. The carbohydrate source was a 1 molar sucrose solution, with 4g of commercially available powdered food colouring and carrying agent mixture (red: Allura red AC, E129, 12.5% pure colour, 2% aluminium. Blue: Brilliant blue FCF, E133, 9.26% pure colour, 3.6% aluminium, carrying agent sulphate / chloride, RBV Birkmann GmbH & Co) per litre of solution. In rats, ingested Brilliant

Blue FCF is not absorbed, and circa 97% of ingested Brilliant Blue FCF is excreted in the faeces [1]. A large proportion of ingested Allura Red is broken down or absorbed by rats after ingestion, but nonetheless 29% of ingested material is excreted in the faeces [2].The coloured carbohydrate source was provided in 1.5 ml centrifuge tubes plugged with cotton wool, and replaced twice weekly. The protein source was adapted from Bhatkar mix [3], and consisted of a mixture of 7g agar, 500 ml water, 1.5 eggs and 2g of powdered food colouring. A centrifuge cap full (c. 250 µl) of the protein feed was provided twice per week. In half of the colonies the sugar was coloured red and the protein blue, and in the other half of the colonies these colours were reversed. Uncoloured water was provided *ad libitum* in a similar manner to the sugar solution. The plaster nest was moistened twice per week, and water tubes refilled as necessary. Colonies were maintained at 21 c° at a 12:12 light:dark cycle.

Once per week the opaque lid was removed from each nest, and the entire foraging box was photographed using a digital camera (Canon PowerShot SX170 IS). After two months the ants were returned to their mother colonies, and the final state of the nest was photographed.

Description of 'toilet' locations

So as to objectively describe the location of the putative 'toilets', a person unconnected with the study was asked to localise the location of all "dark patches" in each nest. Each dark patch was assigned a cavity (top left, top right, bottom left, bottom right, or nest entrance), an XY coordinate within the cavity (with higher numbers being further from the nest entrance), and a square type (corner, side, middle, cavity entrance, or nest entrance) (see figure 1). For the complete instructions provided to this observer see below. The observed count of locations of the 'toilets' were compared to the expected null hypothesis of a random distribution, based on square type frequency, using a chi squared test.

Below is the instruction sheet provided to the uninformed participant used to localise the faecal patches in the manuscript:

nest analysis instructions

You will be presented with 21 artificial ant nests.
You will also be presented with a transparent grid the same shape as the ant nest.
The layout of the ant nests is show in figure 1, below.
The ant nest may, or may not, have one or more darker or coloured patches.

For each dark patch, find the point which best describes the patch (darkest/most intense/origin).

For this point, and using the transparent grid, fill out in the datasheet using figure 1:

1. What colony the point is in (the letter next to the dot).
2. What cavity the point is in (TL, TR, BL, BR)
3. What square type it is (C, S, M, E, or NE).
4. What its X coordinate is (0, 1, 2, 3, or 4).
5. What its Y coordinate is (0, 1, 2,3, or 4).
6. How many dark patches there are in the nest.

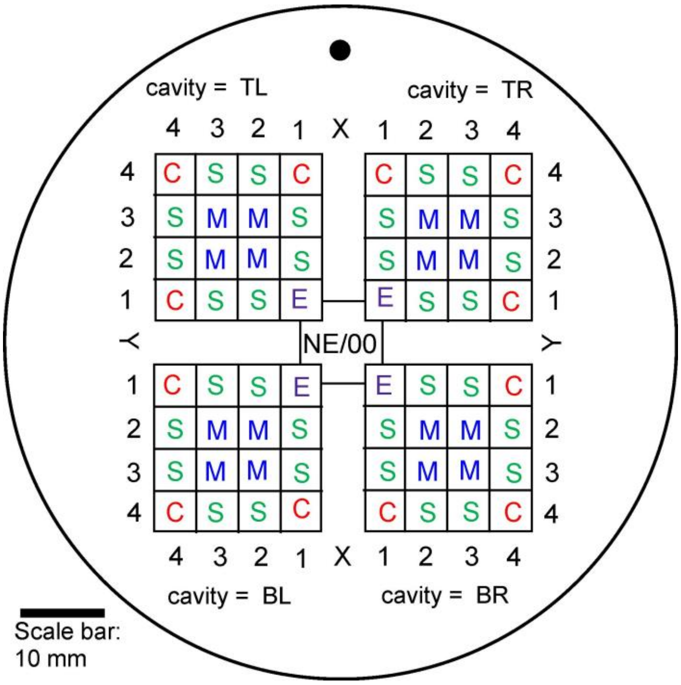

Figure 1 – a plan of an artificial ant nest. Each square of the grids is $5mm^2$.

S1 Methods References

1. Aguilar F, Dusemund B, Galtier P, Gilbert J, Gott DM, et al. (2010) Scientific Opinion on the re-evaluation of Brilliant Blue FCF (E 133) as a food additive. Eur Food Saf Auth J 8: 1854.

2. White RG (1970) Metabolic fate of orally ingest ed non-toxic Red Z-4576. Unpubl Rep 21855 Buffalo Res Lab.

3. Bhatkar A, Whitcomb WH (1970) Artificial diet for rearing various species of ants. Fla Entomol 53: 229–232.
